# Glioblastoma stem cell–immune microenvironment crosstalk: molecular mechanisms and therapeutic opportunities

**DOI:** 10.3389/fimmu.2026.1972420

**Published:** 2026-10-09

**Authors:** Chuanhai Xu, Linlin Chai, Wei Yuan

**Affiliations:** 1 Department of Pathology, Yancheng Clinical Medical College of Jiangsu University, The First People’s Hospital of Yancheng, Yancheng, Jiangsu, China; 2 Department of Translational Medicine Centre, The Fourth Affiliated Hospital of Nantong University, Yancheng, Jiangsu, China; 3 School of Life Science, Nantong Laboratory of Development and Diseases, Key Laboratory of Neuroregeneration of Jiangsu and Ministry of Education, Co-innovation Centre of Neuroregeneration, Nantong University, Nantong, Jiangsu, China

**Keywords:** glioblastoma, glioblastoma stem cells, immune escape, precision therapy, treatment resistance, tumour immune microenvironment, tumour–immune crosstalk

## Abstract

Glioblastoma (GBM) remains one of the most aggressive and treatment-resistant primary brain malignancies, with limited improvement in long-term survival despite advances in multimodal therapy. Glioblastoma stem cells (GSCs) are central to tumour initiation, recurrence and therapeutic resistance, but their malignant properties are not determined by intrinsic programmes alone. Increasing evidence indicates that reciprocal interactions between GSCs and the tumour immune microenvironment (TIME) establish a dynamic ecosystem that promotes stemness, immune escape and treatment resistance. In this review, we summarise the molecular and cellular mechanisms underlying GSC – TIME crosstalk, with emphasis on tumour-associated macrophages, myeloid-derived suppressor cells, T cells, regulatory T cells, natural killer cells, B cells, plasma cells and neutrophils. We highlight major signalling and metabolic networks, including B2M/TGF-β1, IL8/CXCR2, IFI35/NF-κB, lactate/lactylation, IgG/FcγRIIA and m6A/LOXL2 signalling, as well as extracellular vesicle-mediated communication. We further discuss how these interactions sustain GSC stemness, facilitate metabolic adaptation, promote immune escape and contribute to resistance to radiotherapy, temozolomide and targeted therapies. Emerging therapeutic approaches—including tumour-associated macrophage/myeloid-derived suppressor cell (TAM/MDSC) reprogramming, metabolic and epigenetic intervention, immune checkpoint blockade, bispecific antibodies, chimeric antigen receptor T/natural killer (CAR-T/NK) cell therapy and extracellular vesicle-based delivery—are also evaluated. Finally, we discuss the importance of spatial heterogeneity, blood-brain barrier limitations and rational combination strategies. Understanding GSC-TIME crosstalk may provide a framework for developing more effective and biologically informed precision therapies for GBM.

## 1 Introduction

Glioblastoma (GBM) is the most aggressive primary malignant tumour of the adult central nervous system, with an annual incidence of approximately 3.2 cases per 100,000 people. It accounts for about 56% of gliomas and 14% of primary brain tumours (1, 2). Despite advances in surgery, radiotherapy and systemic therapy, the prognosis remains poor. Since the introduction of temozolomide (TMZ)-based chemoradiotherapy (Stupp regimen) in 2005, median overall survival has increased only modestly, from approximately 12 to 15 months, whilst the 5-year survival rate remains below 10% (3). Although anti-angiogenic agents such as bevacizumab provide clinical benefit in selected settings, their impact on overall survival remains limited (4). Thus, identifying the biological mechanisms underlying GBM progression, recurrence and treatment resistance remains an important unmet need.

Glioblastoma stem cells (GSCs) provide an important framework for understanding the remarkable heterogeneity and therapeutic resistance of GBM (5). Characterised by self-renewal, multilineage differentiation and resistance to radiotherapy and chemotherapy, GSCs contribute substantially to tumour initiation, progression and recurrence. Importantly, GSCs do not function autonomously but actively remodel their surrounding microenvironment to establish niches that support their survival and stemness (5). Increasing evidence indicates that GBM is not an immunologically silent tumour. Instead, its tumour immune microenvironment (TIME) contains diverse immune populations that are extensively reprogrammed towards immunosuppressive states by tumour-derived signals (6, 7). Reciprocal communication between GSCs and immune cells through cytokines, chemokines, growth factors, metabolites and extracellular vesicles enables GSCs to suppress anti-tumour immunity whilst simultaneously acquiring survival and stemness signals from the microenvironment (8). This bidirectional crosstalk provides an important mechanistic basis for GBM progression and therapeutic resistance.

Recent advances in single-cell and spatial technologies have substantially improved the resolution with which GSC–TIME interactions can be investigated. Single-cell RNA sequencing (scRNA-seq) has revealed distinct transcriptional states of GSCs and infiltrating immune populations, whereas spatial transcriptomics and multiplex imaging platforms, including 10x Visium, CODEX and MIBI, have defined their spatial organisation and potential cell–cell interactions (9). In parallel, CRISPR-based functional genomic screening has enabled systematic identification of genes involved in GSC stemness, immune evasion and therapeutic resistance (10). Against this background, this review summarises the major signalling networks linking GSCs with the principal components of the TIME, examines how these interactions promote GBM progression, stemness, treatment resistance and immune escape, and discusses emerging therapeutic strategies for disrupting GSC-TIME crosstalk.

## 2 Biological characteristics of glioblastoma stem cells

### 2.1 Discovery and definition of GSCs

The concept of GSCs emerged from studies of brain tumour stem-like populations in the late 1990s, when neural progenitor-like cells were isolated from human brain tumours (11). Between 2002 and 2004, several groups identified highly tumourigenic, self-renewing GBM cell populations capable of reproducing the parental tumour phenotype in immunodeficient mice, establishing the GSC concept (5, 12). Singh et al. subsequently demonstrated that CD133 (Prominin-1)-positive GBM cells possessed enhanced tumourigenic potential, establishing CD133 as an important, although not exclusive, GSC marker (13). CD133-positive GSCs also display increased resistance to radiotherapy and chemotherapy, partly through enhanced DNA repair and anti-apoptotic mechanisms (14).

GSCs are now recognised as a heterogeneous population that cannot be defined by a single marker. Transcription factors including SOX2 and OLIG2 are central regulators of stemness, self-renewal and therapeutic resistance (5, 15). OLIG2 can sustain GSC proliferation and stemness through regulation of EGFR signalling and cell-cycle programmes (16). Other markers and regulators, including NESTIN, BMI1, LIN28A and NOTCH1, have also been associated with GSC identity (5). Importantly, single-cell analyses demonstrate substantial phenotypic and transcriptional heterogeneity amongst GSCs, with different populations relying on distinct combinations of markers and regulatory programmes (17). Thus, GSC identity is better viewed as a dynamic cellular state than as a fixed population defined by a single marker.

### 2.2 Heterogeneity and molecular states of GSCs

GBM exhibits extensive molecular and cellular heterogeneity, which is particularly evident amongst GSC populations. Transcriptomic analyses originally classified GBM into Classical, Mesenchymal, Proneural and Neural subtypes (18). Although the Neural subtype has subsequently been questioned as a distinct biological entity, the classification provided an important framework for understanding GBM heterogeneity (5). Classical tumours are frequently characterised by EGFR amplification and strong proliferative programmes, whereas Mesenchymal tumours commonly show NF1 alterations and enhanced invasive and inflammatory features (19). GSCs associated with different molecular states consequently display distinct patterns of proliferation, invasion and immune interaction (20, 21).

GSC heterogeneity is also dynamic. Single-cell and spatial transcriptomic studies indicate that GSCs can transition between quiescent, proliferative and intermediate states in response to developmental and microenvironmental signals (21). The concept of stemness as a continuous state or “stemness entropy” rather than a binary property further emphasises the plasticity of GSCs (22). This dynamic state transition may explain why GBM can develop reversible, non-genetic therapeutic resistance even without the acquisition of new driver mutations.

### 2.3 Molecular mechanisms maintaining GSC stemness

Several developmental and inflammatory signalling pathways, including Notch, SHH, WNT/β-catenin and NF-κB, contribute to GSC maintenance. Notch signalling activates stemness-associated genes such as HES1 and HEY1 following ligand-dependent NICD nuclear translocation (23). G9a-mediated epigenetic regulation can further enhance Notch activity and GSC tumourigenicity. SHH–GLI signalling promotes GSC self-renewal and has been associated with GBM invasion and recurrence (24), whilst persistent NF-κB activation supports inflammatory signalling and stemness (25). Other regulators, including NR4A2, MEOX2 and TAGLN, link lipid metabolism, extracellular matrix remodelling and hypoxic adaptation to GSC maintenance (26–28).

Epigenetic regulation provides an additional layer of stemness control. GSCs exhibit widespread DNA methylation abnormalities, including promoter hypermethylation of differentiation-associated genes (29). Histone modifications such as H3K27ac, H3K4me3 and H3K27me3 establish distinct chromatin states associated with stemness and differentiation (30). RNA modifications, particularly N^6^-methyladenosine (m^6^A), further regulate GSC self-renewal, differentiation and therapeutic response (31).

Metabolic plasticity is another defining characteristic of GSCs. Although GSCs can rely heavily on glycolysis, they retain substantial metabolic flexibility and can adapt oxidative phosphorylation and other pathways according to environmental conditions (32). Cholesterol metabolism contributes to stemness through SREBP2-dependent biosynthesis and Sonic hedgehog signalling, whilst glutamine metabolism supplies tricarboxylic acid cycle intermediates and substrates for epigenetic regulation (33). Mitochondrial plasticity further enables GSCs to withstand metabolic stress (34). Microenvironment-derived metabolites also contribute to this process; for example, cholesterol supplied by lymphatic endothelial cells can be incorporated by GSCs, illustrating the metabolic coupling between tumour cells and their niche. PTEN C211 succinylation provides an additional metabolic–epigenetic regulatory mechanism involving Fe-S cluster homeostasis and genomic stability (35).

Hypoxia is a major extrinsic regulator of GSC maintenance. Activation of HIF-1α/HIF-2α induces glycolytic, angiogenic and stemness-associated programmes, including GLUT1, LDHA, VEGF, OCT4 and NANOG (34). Hypoxia can also promote dedifferentiation towards a more primitive stem-like state (36). Translation-associated factors such as YRDC contribute to GSC adaptation to hypoxic stress (37). In parallel, extracellular vesicles provide an additional communication route between GSCs and the surrounding microenvironment. GSC-derived exosomes can carry miRNAs, proteins and non-coding RNAs, including miR-21, miR-29, miR-221, PD-L1, EGFRvIII, and MALAT1, which can modulate TAM polarisation, MDSC function and T-cell exhaustion (38–43). These findings indicate that stemness is maintained through an integrated network involving transcriptional, epigenetic, metabolic and microenvironmental signals.

GSCs also hijack developmental signalling networks of the neural stem cell (NSC) niche. In the normal brain, NSCs are concentrated mainly in the subventricular zone and subgranular zone of the hippocampus, where developmental signals regulate neural stemness and differentiation (7). GSCs exploit several of these developmental pathways, particularly Notch, SHH and WNT, to establish a tumour-supportive niche (44). Neurodevelopmental transcription factors such as SOX2, OLIG2 and PAX6 remain highly active in GSCs and drive developmental gene programmes (5). BMP signalling illustrates the context-dependent nature of these pathways: although BMP signalling generally promotes differentiation of normal NSCs, its dysregulation in GBM may instead support GSC maintenance or immunosuppressive microenvironmental remodelling (45). Similarly, EGF and FGF, which normally regulate NSC proliferation, are frequently activated in GSCs and promote self-renewal (46). GSCs can therefore be viewed as exploiting and remodelling developmental signalling networks to establish a tumour-adapted stem cell niche.

## 3 Composition of the tumour immune microenvironment

### 3.1 Immune cell composition

The TIME of GBM is a complex and dynamic ecosystem whose cellular composition differs substantially from that of peripheral blood and normal brain tissue. Enrichment of immunosuppressive populations, including tumour-associated macrophages (TAMs), myeloid-derived suppressor cells (MDSCs) and regulatory T cells (Tregs), together with dysfunction of effector cells such as CD8+ T cells and NK cells, contributes to immune escape (7). Understanding the cellular composition and spatial organisation of TIME is therefore essential for defining GSC–immune-cell crosstalk.

TAMs, comprising both resident microglia and infiltrating monocyte-derived macrophages, are the predominant immune population in GBM and may account for approximately 30–50% of the tumour mass or immune compartment, depending on the analytical method (47). Rather than representing discrete M1 and M2 states, TAMs exhibit substantial phenotypic and functional plasticity and occupy a continuum of activation states. Resident microglia and monocyte-derived macrophages also display distinct spatial distributions and transcriptional programmes (48). Nevertheless, immunosuppressive TAM states are prominent in GBM and contribute to angiogenesis, invasion and immune suppression through factors including VEGF, PDGF, MMPs, IL-10 and TGF-β. TAM-mediated inhibition of T-cell activity involves both soluble mediators and immune-regulatory molecules such as PD-L1. Emerging studies further implicate non-coding RNAs and TREM2 signalling in GSC–TAM communication. For example, circPRKD3 regulates TAM polarisation through a STAT3-dependent mechanism, highlighting the contribution of tumour-derived RNA programmes to myeloid reprogramming (49). TREM2 may also regulate the balance between immunosuppression and phagocytic activity in tumour-associated myeloid cells, suggesting that therapeutic targeting will require careful consideration of context and spatial heterogeneity (50).

MDSCs constitute another major immunosuppressive population in GBM. They comprise heterogeneous immature myeloid cells that are broadly classified into PMN-MDSCs and M-MDSCs according to phenotype and morphology (51). In humans, PMN-MDSCs are minimally defined as CD11b+CD14−CD15+ (or CD66b+), whereas M-MDSCs are defined as CD11b+CD14+CD15−HLA-DR^low/neg^. CD33 is broadly expressed by both MDSC subsets and mature monocytes; it may be used as a supportive myeloid marker, but it is neither required for M-MDSC definition nor sufficient to distinguish M-MDSCs from monocytes. Phenotypic classification should therefore be complemented, where possible, by functional evidence of T-cell suppression (51). MDSCs accumulate in peripheral blood and tumour tissue and suppress anti-tumour immunity through ARG1, iNOS, reactive oxygen species (ROS) and reactive nitrogen species (RNS), as well as immune checkpoint and cytokine-mediated mechanisms (52, 53). ARG1-mediated depletion of L-arginine impairs CD3ζ expression and T-cell receptor signalling, whilst NO generated by iNOS further compromises T-cell proliferation and function. MDSCs can also promote Treg expansion and suppress NK-cell activity. Spatial and single-cell studies suggest that specific MDSC populations preferentially localise near GSC-rich regions, supporting the existence of direct tumour–myeloid communication. Beyond these classical definitions, two emerging populations warrant attention in GBM. Early-stage MDSCs (E-MDSCs; Lin−HLA-DR−CD33+CD11b+CD14−CD15−) represent immature progenitors that are difficult to capture with conventional panels; single-cell and spatial analyses of human IDH-wildtype GBM have identified a prominent E-MDSC compartment that is recruited by glioma stem-like cell-derived chemokines and, in turn, secretes tumour-supportive factors, positioning these cells as direct participants in GSC–myeloid crosstalk (51, 54). In parallel, nonclassical immunosuppressive monocytes—phenotypically CD11b+CD15−HLA-DR+CD16+, with subsets expressing PD-1/PD-L1—are induced by GBM-derived extracellular vesicles and suppress T-cell responses (55, 56). Because these HLA-DR+CD16+ cells are phenotypically distinct from conventional M-MDSCs, they should not be classified as M-MDSCs on phenotype alone; these overlapping populations may nevertheless explain part of the heterogeneity observed across studies.

T cells exhibit marked functional impairment in the GBM TIME (7). CD8+ T cells, although central to anti-tumour immunity, frequently display an exhausted phenotype characterised by expression of PD-1, TIM-3 and LAG-3 and reduced cytotoxic activity (57). Tregs are enriched amongst CD4+ T cells and suppress anti-tumour responses through CTLA-4 and immunoregulatory cytokines, including IL-10 and TGF-β (58). Their enrichment is associated with an immunosuppressive tumour milieu and may be particularly prominent in perivascular and tumour-core regions. γδ T cells are also detected in GBM, although their functional contribution remains incompletely defined.

NK cells provide an additional component of innate anti-tumour immunity but are functionally suppressed in the GBM TIME (7). GSCs and other tumour cells can evade NK-cell recognition through altered MHC-I expression and reduced expression of activating NKG2D ligands (59). TGF-β and other suppressive mediators produced by TAMs, MDSCs and tumour cells further reduce NK-cell IFN-γ production and cytotoxicity. GSCs may also express non-classical MHC molecules such as HLA-G, providing additional inhibitory signals.

B cells and plasma cells represent a relatively understudied component of the GBM TIME. Although generally less abundant than myeloid cells, recent single-cell and spatial studies suggest that B-cell and plasma-cell infiltration may have context-dependent associations with clinical outcome (7, 60). CD20+ B cells and CD138+ plasma cells show distinct spatial distributions, with plasma cells frequently accumulating around blood vessels and necrotic regions. Emerging evidence involving IgG–Fc receptor signalling suggests that humoral immunity may influence GSC biology in addition to conventional anti-tumour responses.

### 3.2 Tumour-associated astrocytes

Tumour-associated astrocytes (TAAs) constitute an important non-immune component of the GBM TIME. Operationally, we define TAAs as non-haematopoietic, non-endothelial glial cells located within or immediately adjacent to the tumour mass that acquire a tumour-induced transcriptional and functional state, typically involving JAK/STAT3 and NF-κB activation and the secretion of immunoregulatory and trophic factors in response to TGF-β, hypoxia and other microenvironmental cues. Markers commonly used for astrocyte or glial-cell characterisation, including GFAP, S100β, SOX9 and ALDH1L1, are not TAA-specific; SOX9 in particular may also be expressed by GSCs. No validated TAA-specific marker currently exists. TAA identity should therefore be assigned through the combined assessment of spatial proximity to tumour cells, exclusion of haematopoietic and endothelial lineages, and functional evidence of tumour-associated reprogramming (61–65). In contrast to generic reactive astrocytes elicited by acute, non-neoplastic CNS injury, TAAs are characterised by sustained spatial and functional coupling to tumour cells and a net contribution to immunosuppression and tumour support rather than to glial scar formation or tissue repair (61–64). Rather than functioning solely as passive bystanders, these astrocytes respond to tumour-derived signals and acquire phenotypes that promote tumour progression and immune suppression (65). TGF-β, hypoxia and other microenvironmental signals contribute to this phenotypic reprogramming.

TAAs secrete factors including CHI3L1, TGF-β and IL-6 that can modulate immune-cell function and support tumour growth (61–63). CHI3L1 has been implicated in suppression of T-cell proliferation and IFN-γ production (66, 67). TAAs may also express immune-regulatory molecules, including PD-L1 (68), although the extent and functional significance of these pathways require further validation. Conversely, TAA-derived VEGF-A and other trophic signals can support GSC survival and growth, whilst GSC-derived cues, including TGF-β, may reinforce the tumour-associated phenotype of astrocytes (64). At the invasive margin, interactions amongst TAAs, neurons and GSCs may additionally regulate glutamate homeostasis and protect tumour cells from excitotoxic stress, thereby facilitating invasion (69).

### 3.3 Spatial organisation of GSC–immune niches

GBM TIME exhibits pronounced spatial heterogeneity that reflects regional differences in oxygenation, vascular architecture, extracellular matrix composition and tumour-cell states (47). Spatial transcriptomic analyses indicate that immune cells occupying different anatomical niches acquire distinct transcriptional programmes, demonstrating that immune phenotypes are strongly shaped by local environmental cues.

Pseudopalisading regions surrounding necrotic cores are characterised by severe hypoxia and HIF-1α activation (54, 70). TAMs and MDSCs are enriched in these regions, whereas cytotoxic T cells are relatively sparse. Hypoxia, TGF-β and metabolic stress collectively favour an immunosuppressive niche (6).

The perivascular region represents another critical immune and stemness niche (71). Endothelial cells can maintain GSC self-renewal through Notch ligands such as JAG1 and DLL4 and chemokines such as CXCL12 (72, 73). Although GBM vessels are abnormal and highly permeable, perivascular areas support GSC maintenance and provide an important route for immune-cell trafficking. Tregs frequently accumulate around tumour-associated vessels, whilst B cells and plasma cells may also form local aggregates (74). These observations suggest that the perivascular niche represents an important interface between vascular, immune and stem-cell programmes (71).

The tumour core generally contains fewer functional effector immune cells because of high tumour-cell density, hypoxia, necrosis and immunosuppressive signalling. GSCs in this compartment may adopt relatively quiescent states associated with enhanced treatment resistance (75). By contrast, the invasive margin exhibits greater cellular and molecular plasticity, with increased interaction between tumour cells and the surrounding normal brain. GSCs can form synapse-like connections with neurons and astrocytes, and neurotransmitter signalling, including glutamate-mediated communication, can promote tumour-cell proliferation and invasion (69). Despite relatively greater immune-cell infiltration, GSCs can maintain immune evasion through checkpoint expression and other suppressive mechanisms (75).

Recent evidence further suggests that skull bone marrow-derived immune cells can access the brain through skull-meningeal communication routes and contribute to local immune organisation (76). The formation of tertiary lymphoid structures (TLS) has been reported in GBM and may be associated with enhanced anti-tumour immunity and improved response to immunotherapy (74). These findings highlight the importance of considering where immune cells are located, rather than simply measuring their overall abundance. Collectively, these findings define a spatially compartmentalised GSC–immune landscape in GBM, in which the necrotic core, pseudopalisading/hypoxic zone, tumour core, perivascular niche and invasive margin contain distinct immune programmes and GSC states. Skull bone marrow-derived myeloid cells may further contribute to this organisation through skull-meningeal channels (**Figure 1**).

**Figure 1:**
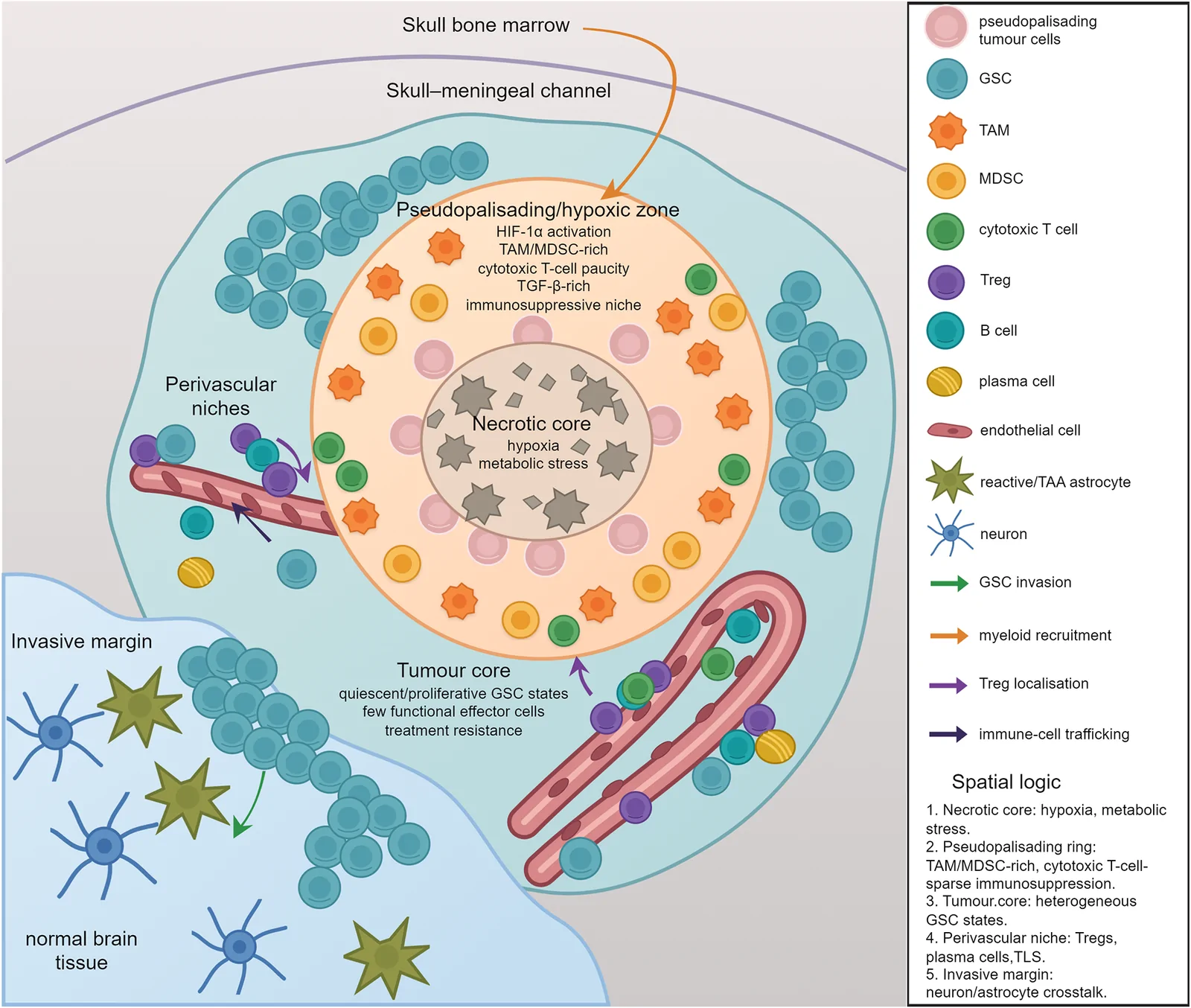
Spatial organisation of GSC–immune niches in glioblastoma. The schematic illustrates the spatial relationship between glioblastoma stem cells (GSCs) and immune populations in the glioblastoma tumour immune microenvironment (TIME). The necrotic core is characterised by hypoxia and metabolic stress. The surrounding pseudopalisading/hypoxic zone shows HIF-1α activation, enrichment of tumour-associated macrophages (TAMs) and myeloid-derived suppressor cells (MDSCs), cytotoxic T-cell paucity, and a TGF-β-rich immunosuppressive niche. The tumour core contains quiescent and proliferative GSC states, few functional effector cells, and treatment-resistant populations. Perivascular niches contain accumulating Tregs and vascular-associated B-cell/plasma-cell aggregates, whereas tertiary lymphoid structure (TLS) formation has been reported in GBM. The invasive margin shows crosstalk amongst GSCs, neurons and tumour-associated astrocytes (TAAs). Skull bone marrow-derived cells may enter through skull-meningeal channels. Arrows indicate GSC invasion, myeloid recruitment, Treg localisation and immune-cell trafficking.

## 4 Signalling crosstalk between GSCs and immune cells

### 4.1 Interaction between GSCs and TAMs

GSC–TAM communication represents one of the best-characterised components of the GBM TIME (77). The interaction is bidirectional: GSCs recruit and reprogramme myeloid cells, whilst TAM-derived cytokines, growth factors and metabolic signals reinforce GSC stemness, survival and invasion. The major signalling axes mediating GSC-immune crosstalk are summarised in **Figure 2**.

**Figure 2:**
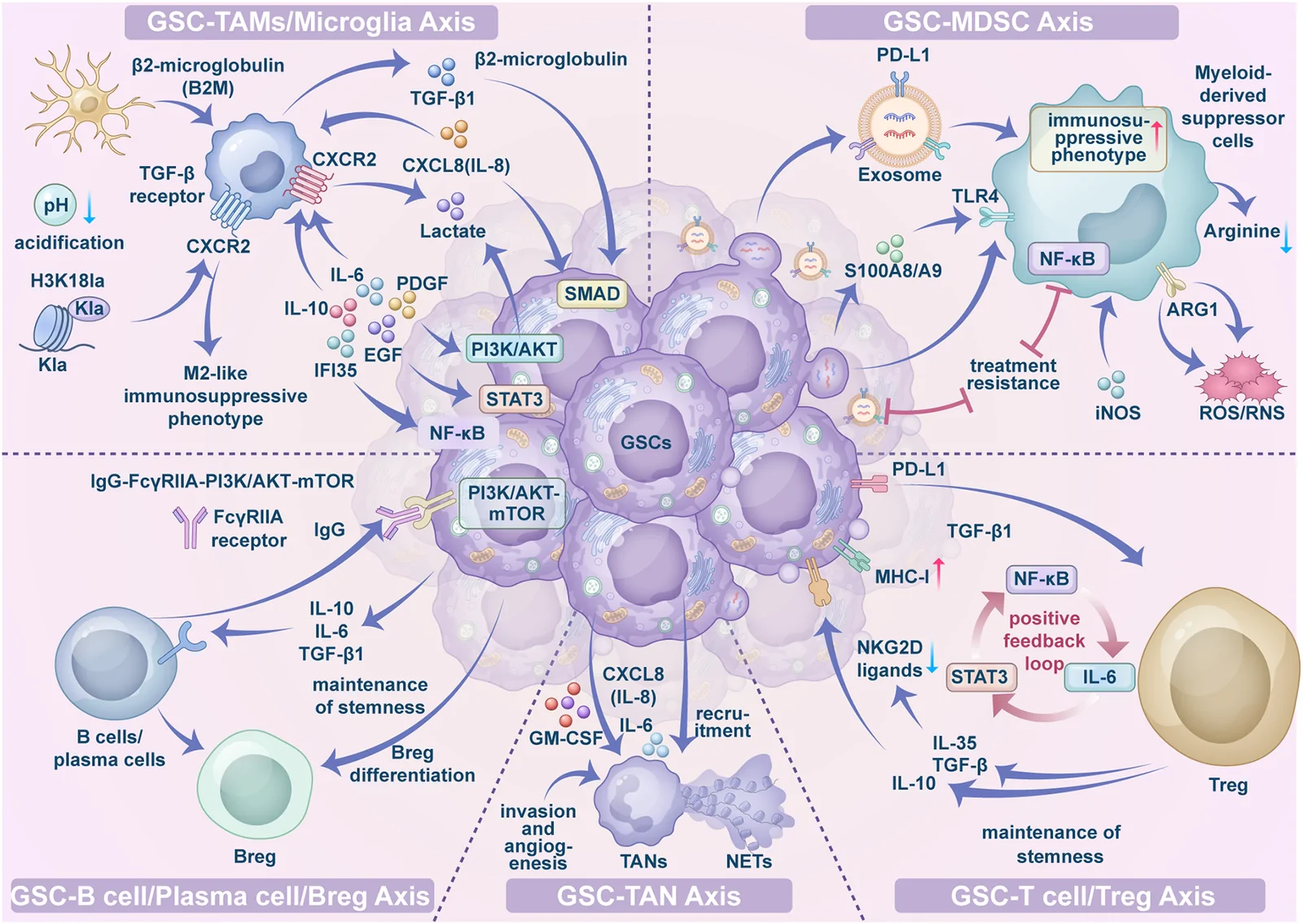
Bidirectional signalling crosstalk between GSCs and immune components of the TIME. Centre: GSC cluster. Surrounding panels illustrate the following interactive axes: GSC–tumour-associated macrophages (TAMs)/microglia, GSC–myeloid-derived suppressor cells (MDSCs), GSC–T/NK cells/regulatory T cells (Tregs), GSC–B cells/plasma cells/regulatory B cells (Bregs), and GSC–tumour-associated neutrophils (TANs), highlighting key ligand–receptor pairs, downstream intracellular effectors, and associated functional outcomes.

The B2M/TGF-β1 axis represents one mechanism linking GSCs to TAM-mediated immunosuppression. GSC-derived B2M has been reported to enhance TGF-β/SMAD signalling and promote an immunosuppressive myeloid phenotype (77). TGF-β1 subsequently suppresses T-cell and NK-cell activity and may reinforce GSC stemness. B2M-associated signalling has also been linked to PI3K/AKT activation in GSCs, suggesting that this pathway may simultaneously regulate tumour-cell fitness and immune suppression.

The CXCL8 (IL-8)/CXCR2 axis provides a complementary mechanism for myeloid-cell recruitment (78). GSC-derived IL-8 attracts CXCR2-expressing myeloid populations, including MDSCs and subsets of TAMs, and may promote their immunosuppressive functions. This pathway therefore links chemotaxis with functional myeloid reprogramming and represents a potential therapeutic target.

IFI35-mediated non-canonical NF-κB signalling represents another emerging GSC–TAM regulatory mechanism (25). Mechanistically, IFI35 functions as a scaffold protein that binds both the E3 ubiquitin ligase TRIM21 and NFKB1, promoting TRIM21-dependent ubiquitination and proteasomal processing of the p105 precursor into the DNA-binding subunit p50. p50 preferentially heterodimerises with RELB, and the resulting RELB/p50 complex is enriched at the promoters of chemokine and cell-migration genes, activating their transcription in a cell-autonomous manner and thereby driving paracrine recruitment and maintenance of immunosuppressive TAMs. However, therapeutic targeting of IFI35 remains experimental.

Metabolic communication provides an additional layer of GSC–TAM interaction. GSC-derived lactate can acidify the tumour microenvironment and influence macrophage function. Lactate also serves as a substrate for protein lactylation, including histone H3K18 lactylation, which has been implicated in macrophage polarisation (79). Lactylation may additionally influence expression of phagocytosis-regulating molecules such as CD47, linking tumour metabolism with immune evasion (80).

Other emerging pathways include TNFAIP6/EGFR signalling (81), WTAP/m6A/LOXL2-mediated extracellular matrix and myeloid reprogramming (82), and ICAM1/β-catenin/PD-L1 signalling (83). Mesenchymal stem/stromal cells may further amplify these interactions by releasing IL-6, IL-8, VEGF and extracellular vesicles (84, 85). Collectively, these findings indicate that GSC–TAM communication is not mediated by a single pathway but by an interconnected network involving cytokines, chemokines, metabolism, RNA modification and cell adhesion.

Innate immune sensing intersects with this signalling landscape through inflammasome activation. NLRP3 inflammasome assembly and consequent IL-1β/IL-18 release have been observed in GBM, where NLRP3 signalling expands the granulocytic MDSC compartment and reinforces an immunosuppressive myeloid stroma; notably, NLRP3 activation can antagonise the therapeutic benefit of STING agonism in GBM models (86). These findings indicate context-dependent roles for NLRP3: inflammasome activation can expand granulocytic MDSCs and antagonise STING-mediated therapy in GBM models (86), whereas NLRP3 is required for restored T-cell function after TIGIT knockdown in human EV-conditioned myeloid cells (87). Pharmacological NLRP3 inhibition (e.g., MCC950) should therefore be evaluated according to the relevant checkpoint context rather than assumed to be uniformly beneficial.

### 4.2 Interaction between GSCs and MDSCs

GSC-MDSC communication depends on chemokines, inflammatory mediators and extracellular vesicles. Single-cell and spatial transcriptomic data from IDH-wildtype GBM reveal two MDSC populations: an early progenitor population (E-MDSC) and a monocytic population (M-MDSC) (54). Ligand-receptor analyses show that glioma stem-like cells secrete chemokines that attract E-MDSCs; these cells then produce growth and survival factors for tumour cells. Glioma-derived CCL2 and CCL7 also recruit immunosuppressive CCR2+/CX3CR1+ M-MDSCs into the tumour microenvironment. Combined neutralisation of both chemokines blocks the migration of CCR2-expressing cells (88). In resistant murine gliomas, CCR2 inhibition lowers tumour-associated MDSC numbers and improves the survival benefit of anti-PD-1 therapy. These results confirm that this recruitment axis is functionally relevant (89). Single-cell and spatial analyses have additionally identified S100A9-high immunosuppressive monocytes in GBM; these cells are spatially associated with immunosuppressive macrophages and exhausted CD8+ T cells and correlate with poor prognosis (90).

GSC-derived exosomes promote an immunosuppressive PD-L1+ myeloid phenotype in human monocytes, partly through STAT3-pathway components (38). They also induce CD14+ monocytic cells with a M-MDSC-like phenotype that suppresses T-cell activation (39). Broader glioma-EV studies support this model. GBM small EVs carrying LILRB2 promote MDSC formation and expansion (91). VEGF-A packaged in GBM exosomes drives MDSC differentiation and increases MDSC-derived TGF-β1 secretion (92). Several glioma exosomal non-coding RNAs exert similar effects. Hypoxia-induced glioma exosomes deliver miR-29a and miR-92a, which expand functional MDSCs through Hbp1 and Prkar1a (93). Hypoxic glioma exosomes also use miR-10a and miR-21 to enhance MDSC expansion and activation (94). Exosomal miR-1246 from glioma patient body fluids drives MDSC differentiation and activation (95), and exosomal lncRNA AGAP2-AS1 promotes MDSC-derived TGF-β1 secretion (96). IFN-γ-exposed GBM EVs additionally increase PD-L1 and IDO1 expression and thus enhance MDSC induction from monocytes (55). EV-associated PD-L1 also contributes to induction of immunosuppressive nonclassical monocytes (56).

Together, these findings show that GSC-MDSC communication is bidirectional. This communication combines chemokine-directed recruitment with exosome-mediated delivery of proteins and non-coding RNAs (97, 98). Hypoxic, MDSC-rich regions may further sustain glioma stemness through hypoxia-inducible factors and chemokine gradients (47). These observations support the view that MDSCs and GSCs form a spatially organised, self-amplifying immunosuppressive niche.

### 4.3 Interaction between GSCs and T/NK cells

GSCs suppress T- and NK-cell function through immune checkpoints, cytokines, metabolic competition and direct cell–cell communication. PD-L1 expression is a major mechanism of GSC-mediated T-cell inhibition (7). It can be induced by inflammatory and hypoxic signals, including IFN-γ/STAT1–IRF1 and HIF-1α-dependent pathways. PD-L1 engagement of PD-1 inhibits TCR signalling and reduces T-cell proliferation and cytotoxicity. GSC-derived extracellular vesicles may further disseminate PD-L1 and reinforce systemic immune suppression.

The clinical failure of PD-1/PD-L1 blockade in phase III trials reflects the redundancy of these mechanisms and GBM-specific barriers rather than failure of a single checkpoint pathway (99). Tregs and MDSCs suppress effector cells, whilst TGF-β and lactate impose additional metabolic and functional constraints. Persistent antigen exposure can drive epigenetic T-cell exhaustion characterised by increased TOX and reduced TCF1/FoxO1 activity (100, 101). The blood-brain and blood-tumour barriers may further restrict immune-cell trafficking (102).

CD47 represents another major GSC immune-evasion mechanism. By engaging SIRPα on macrophages, CD47 suppresses phagocytosis and permits GSC survival (103). Importantly, CD47 should be considered primarily as a **myeloid phagocytosis checkpoint**, rather than as a direct NK-cell checkpoint. Its regulation by lactate-associated epigenetic mechanisms provides an important connection between metabolism and innate immune escape (80).

The TIGIT–CD155 axis provides an additional layer of checkpoint-mediated suppression. Its ligand CD155 (PVR) is expressed on GBM cells and on immunosuppressive regulatory B-cell populations, and engages the inhibitory receptor TIGIT on T and NK cells, competing with the activating receptor CD226. In preclinical GBM models, dual TIGIT and PD-1 blockade enhanced anti-tumour immunity and prolonged survival (104), and patient-level analyses have associated TIGIT/PD-1 pathway activity with clinical outcome (105). CD155-based immunoregulation may be particularly relevant to NK-cell dysfunction in GBM (106). Recent work extends TIGIT from a T/NK-cell checkpoint to a marker of immunosuppressive myeloid reprogramming. High-dimensional immunophenotyping of human GBM surgical aspirates and myeloid cells conditioned with EVs from patient-derived GBM cell lines showed broad, concordant TIGIT expression. In donor-matched co-cultures, TIGIT knockdown reduced the immunosuppressive polarisation of EV-conditioned myeloid cells and improved T-cell function. This effect was NLRP3-dependent: TIGIT and NLRP3 were substantially co-expressed and co-localised, and concurrent NLRP3 knockdown abolished the benefit of TIGIT depletion. TIGIT expression correlated with IL-13, and IL-13 blockade unmasked a pro-inflammatory myeloid phenotype. These findings support a model in which TIGIT sustains an IL-13-high state that restrains NLRP3-associated myeloid inflammation and contributes to T-cell dysfunction in the GBM EV niche (87).

GSCs also promote Treg recruitment and expansion through TGF-β1, IL-10 and CCL2 (107–109). Tregs suppress effector T cells through CTLA-4 and immunoregulatory cytokines, whilst Treg-derived factors may activate NF-κB/IL-6/STAT3 signalling in GSCs (110), thereby reinforcing stemness. Similarly, TGF-β and IL-10 can impair NK-cell IFN-γ production and cytotoxicity. The NFAT1–C3a pathway provides an additional potential regulatory mechanism linking complement signalling with immune regulation in GSC niche (47, 111).

### 4.4 Interactions of GSCs with B cells, plasma cells and neutrophils

The contribution of B cells and plasma cells to GSC biology is increasingly recognised. An emerging mechanism involves IgG/FcγRIIA signalling. Plasma-cell-derived IgG has been reported to interact with FcγRIIA on GSCs and activate downstream PI3K/AKT/mTOR signalling, thereby promoting tumour-cell survival and stemness (112). This finding suggests that humoral immunity may have context-dependent pro-tumour effects in GBM.

GSCs can also influence B-cell phenotypes through IL-10, IL-6, TGF-β1 and extracellular vesicles. These signals may promote regulatory B-cell phenotypes and further suppress effector T-cell responses. Chemokines such as CXCL12 and CXCL13 may additionally regulate B-cell localisation.

The therapeutic implications are complex. Although tumour-reactive antibodies can theoretically mediate complement-dependent cytotoxicity or antibody-dependent cellular cytotoxicity, the immunosuppressive GBM environment may limit these effects. The possibility that endogenous IgG can activate pro-tumour Fc receptor signalling therefore warrants consideration when developing antibody-based therapies.

Tumour-associated neutrophils (TANs) are increasingly recognised as components of the GBM TIME, although their relationship with PMN-MDSCs requires careful phenotypic and functional distinction. GSC-derived IL-8, granulocyte–macrophage colony-stimulating factor (GM-CSF) and IL-6 can promote neutrophil recruitment and activation. TANs may support tumour progression through ROS, MMP-9, VEGF and other factors, whilst NETs have been implicated in tumour invasion and angiogenesis (113, 114).

Radiotherapy may further alter neutrophil recruitment and function, generating a radiation–neutrophil–GSC interaction that contributes to treatment resistance (115). However, the precise molecular mechanisms and therapeutic relevance of TAN heterogeneity remain incompletely defined. For both compartments, the evidence base remains thinner than for myeloid cells: B-cell and plasma-cell associations with outcome are context-dependent across cohorts (60, 74), the phenotypic boundary between TANs and PMN-MDSCs is not standardised, and most functional data derive from murine models; these populations should therefore be interpreted as emerging rather than established components of GSC–TIME crosstalk.

Critical appraisal of the evidence base. The major signalling axes described above, together with their functional consequences, evidence levels and therapeutic development stages, are summarised in **Table 1**. The strength of evidence is uneven: mechanisms such as B2M/TGF-β1 (77), IL-8/CXCR2 (78) and IgG/FcγRIIA (112) are supported by patient-derived GSC models combined with orthotopic xenograft validation, and the IL-8/CXCR2 and CCL2/CCR2 recruitment axes are further corroborated by single-cell and spatial analyses of resected human GBM (54, 88). By contrast, lactylation-associated immune regulation (79, 80), WTAP/m6A/LOXL2 signalling (82) and most extracellular-vesicle mechanisms rest predominantly on *in vitro* assays and murine models, in which species differences in myeloid biology and the absence of a fully competent adaptive immune system may overestimate translatability. Moreover, most GSC–immune co-culture systems do not reproduce the spatial constraints of the human tumour. These limitations should temper mechanistic inferences and underscore the need for validation in patient-derived organotypic models, humanised systems and longitudinal clinical samples.

**Table 1:** Major GSC–immune-cell signaling axes: functional consequences, evidence levels, available inhibitors and therapeutic development stages.

Signalling axis	GSC–immune partners	Functional consequence	Evidence level	Representative agents	Development stage	Key reference(s)
B2M/TGF-β1–SMAD	GSC ↔ TAM	Immunosuppressive TAM reprogramming; stemness reinforcement	Patient-derived GSCs + orthotopic xenografts	Galunisertib (TGF-βRI)	Phase 1b/2a (glioma)	(77, 144)
CXCL8/CXCR2	GSC → TAM/MDSC	Myeloid recruitment; immunosuppressive myeloid reprogramming	Patient-derived GSCs + xenografts	SX-682 (CXCR1/2)	Preclinical (mainly)	(78)
IFI35–TRIM21–NFKB1 (RELB/p50)	GSC → TAM	Promotes chemokine and cell-migration gene transcription; supports immunosuppressive TAM recruitment	Patient-derived GSCs + orthotopic models	None (genetic targeting)	Preclinical	(25)
CCL2/CCL7–CCR2	GSC → M-MDSC	M-MDSC recruitment; anti-PD-1 resistance	Human scRNA-seq + murine models	CCR2 inhibition (no specific agent specified in this review)	Preclinical	(54, 88, 89)
Lactate/H3K18 lactylation–CBX3	GSC/TAM metabolism	Macrophage polarisation; CD47 regulation; immune evasion	Preclinical (*in vitro*/*in vivo*)	LDHA (FX11), MCT1/4 (syrosingopine) inhibitors	Preclinical	(79, 80)
IgG–FcγRIIA–PI3K/AKT/mTOR	Plasma cell → GSC	GSC survival/stemness; chemoresistance	Patient-derived specimens + xenografts	FcγR-pathway modulation (concept)	Preclinical	(112)
WTAP/m6A/LOXL2	GSC → ECM/myeloid	ECM remodelling; myeloid reprogramming	Preclinical	None specified in this review	Preclinical	(82)
EV-miRNA/protein cargo (miR-21/29a/92a/1246, LILRB2, PD-L1)	GBM/glioma cell → MDSC/TAM/monocyte	MDSC expansion; immunosuppressive myeloid reprogramming; systemic suppression	*In vitro* + patient biofluids	GW4869; engineered EVs	Preclinical	(38–43, 54–56, 91–96)
PD-L1/PD-1	GSC/TAM → T cell	T-cell inhibition and exhaustion	Human specimens; phase 3 trials negative	Nivolumab	Phase III completed (negative)	(7, 99, 150–152)
CD47/SIRPα	GSC → macrophage	Phagocytosis blockade	Patient-derived GSC xenograft models; early-phase brain tumour study	Magrolimab; next-generation anti-CD47 agents	Phase 1 (PNOC025, brain tumours)	(103, 153)
TIGIT/CD155	GBM/Breg → T/NK cell	T/NK dysfunction; association with patient outcome	Preclinical models + patient association	Anti-TIGIT antibodies	Preclinical in GBM	(87, 104–106)
TIGIT–IL-13–NLRP3	GBM EV → myeloid cell → T cell	TIGIT supports immunosuppressive polarisation; NLRP3 is required for restored T-cell function after TIGIT knockdown; IL-13 blockade reveals a pro-inflammatory phenotype	Human GBM aspirate immunophenotyping + patient-derived GBM cell-line EVs + donor-matched myeloid/T-cell co-culture	Anti-TIGIT; context-dependent NLRP3 modulation	Preclinical	(87)
NLRP3 inflammasome	Myeloid ↔ GSC niche	IL-1β/IL-18 release; Gr-MDSC expansion; antagonises STING agonism	Preclinical (murine)	MCC950 (NLRP3 inhibitor)	Preclinical	(86)

→ Indicates one-way action, ↔ indicates two-way interaction.

## 5 Crosstalk promotes malignant progression

### 5.1 Enhanced stemness maintenance

GSC–TIME crosstalk creates a positive feedback system in which immune-derived signals reinforce GSC stemness, whilst GSCs continuously reshape the microenvironment (116). TAM-derived TGF-β1, IL-6 and IL-10 promote stemness-associated programmes by SMAD, PI3K/AKT and STAT3-related pathways (117–119). EGF and PDGF further activate RTK-dependent PI3K/AKT and RAS/RAF/MEK/ERK signalling (120).

Metabolic interactions also support stemness. GSCs compete with immune cells for glucose, amino acids and lipids, whilst TAM-associated metabolic programmes can provide additional substrates for tumour growth (121). Lipid metabolic reprogramming may therefore represent a shared interface between GSC fitness and myeloid polarisation.

Ferroptosis provides another link between metabolism and GSC survival. GSCs increase resistance to ferroptosis through pathways involving SLC7A11 and GPX4, whereas inflammatory mediators including PGE2 may further enhance this resistance (122). Conversely, pharmacological inhibition of system Xc− or GPX4 can induce ferroptosis and impair GSC survival (47). Cuproptosis, a copper-dependent form of regulated cell death involving lipoylated mitochondrial proteins (123), represents a newer but still largely preclinical therapeutic opportunity (124, 125).

### 5.2 Treatment resistance

GSC–TIME crosstalk contributes to resistance to radiotherapy, TMZ and targeted therapy. TAM-derived TGF-β1 can enhance DNA damage repair (126, 127), whilst lactylation of DNA repair proteins such as Ku70 has been implicated in enhanced double-strand-break repair (128). Hypoxia and TAM accumulation further reinforce radioresistance.

TMZ resistance involves MGMT expression, mismatch-repair defects and increased drug efflux (129–132). TAM-derived IL-6/STAT3 and TGF-β signalling may contribute to MGMT regulation (133), whereas FcγRIIA-associated mTOR signalling has also been implicated in chemotherapy response (112).

Anti-angiogenic therapy illustrates the adaptive nature of GSC–TIME interactions. VEGF blockade can increase hypoxia, promote invasive and stem-like tumour states, and alter TAM and MDSC recruitment (134, 135). EGFR-targeted therapies are similarly limited by molecular heterogeneity and compensatory PI3K/AKT signalling. TAM-derived growth factors may further activate bypass pathways (81).

Mechanical and extracellular matrix signals provide an emerging dimension of treatment resistance. Matrix stiffness and mechanical stress can activate integrin–FAK and YAP/TAZ signalling, thereby influencing GSC stemness and invasion (44, 136). Integration of mechanical, metabolic and immune signals may therefore provide a more complete explanation of GBM aggressiveness.

### 5.3 Immune escape

GSC–TIME crosstalk establishes immune escape through coordinated checkpoint signalling, metabolic suppression and immune-cell reprogramming. PD-L1 expression inhibits infiltrating T cells, whilst extracellular-vesicle-associated PD-L1 may extend this effect beyond direct cell contact (7). CD47 suppresses macrophage-mediated phagocytosis (80). Additional inhibitory receptors, including TIM-3 and LAG-3, contribute to T-cell dysfunction (137, 138), whilst emerging checkpoints such as BTN2A2 may provide additional mechanisms of immune suppression (139).

Metabolic competition further compromises immune function. GSC glycolysis increases extracellular lactate and acidity, which suppress T-cell and NK-cell activity. Depletion of arginine and tryptophan by tumour cells and MDSCs further limits effector-cell function. IDO-mediated tryptophan metabolism and kynurenine accumulation can promote T-cell dysfunction and Treg differentiation (140).

At the same time, GSC-derived TGF-β1, IL-10, PGE2 and lactate reprogramme macrophages, MDSCs and Tregs towards immunosuppressive states (54, 77). This creates a self-reinforcing cycle that maintains the immunologically “cold” phenotype of GBM (141). Conversely, emerging immunomodulators such as IL-19 may partially reverse this state and represent potential therapeutic opportunities (142). Persistent exposure to these suppressive signals ultimately promotes progressive CD8+ T-cell exhaustion, characterised by loss of IFN-γ, TNF-α and IL-2 production and stable epigenetic reprogramming (143).

## 6 Therapeutic strategies targeting GSC–TIME crosstalk

### 6.1 Targeting the GSC–TAM pathway

Because TAMs are central mediators of GSC–TIME communication, targeting myeloid recruitment, survival or functional state represents an attractive therapeutic strategy (53). TGF-β inhibitors, including galunisertib, can interfere with TGF-β-dependent TAM reprogramming and GSC stemness (144). However, the broad biological functions of TGF-β require careful assessment of toxicity and therapeutic window.

CSF-1R inhibition targets TAM survival and differentiation. Agents such as PLX3397 and BLZ945 have reduced TAM accumulation or altered TAM states in preclinical GBM models (145). Clinical experience, however, underscores the difficulty of myeloid depletion: in the phase 2 Ivy Foundation study, PLX3397 (pexidartinib) readily crossed the blood–tumour barrier yet produced a 6-month progression-free survival of only 8.6% with no objective responses in recurrent GBM, with limited histological impact on tumour-associated microglia (146). This outcome illustrates that target engagement and CNS penetration alone do not guarantee efficacy against a plastic myeloid compartment. Their efficacy may be enhanced by combination with radiotherapy or other immunotherapies rather than by monotherapy.

The IL-8/CXCR2 pathway is another potential target because it links GSC-derived chemotaxis to MDSC and myeloid-cell recruitment (78). CXCR2 inhibitors such as SX-682 and IL-8-directed approaches are being investigated mainly in preclinical settings. Similarly, IFI35 inhibition represents an experimental strategy to disrupt GSC-driven non-canonical NF-κB signalling (25).

Overall, therapeutic strategies should move beyond simple depletion of TAMs towards functional reprogramming of myeloid cells, because indiscriminate removal may eliminate potentially beneficial immune functions.

### 6.2 Metabolic intervention strategies

Metabolic vulnerabilities offer opportunities to simultaneously target GSC fitness and immune suppression. Inhibition of lactate production or transport using LDHA or MCT1/4 inhibitors can reduce extracellular acidification and interfere with lactate-dependent signalling (128). Agents such as FX11 and syrosingopine have shown preclinical activity, although their clinical translation remains uncertain.

Lactylation provides a further therapeutic target because lactate availability influences histone and protein lactylation. Strategies under investigation include inhibition of LDHA, lactate transporters and potential lactylation-associated writers such as p300/CBP. These approaches remain experimental.

Cholesterol metabolism is another vulnerability. SREBP2 inhibition, statins and blockade of intracellular cholesterol transport through NPC1 can impair GSC survival and signalling (32, 147). Similarly, glutaminase inhibition with agents such as CB-839/telaglenastat targets the glutamine dependence of GSCs, although compensatory metabolic pathways may limit monotherapy efficacy.

Ferroptosis induction using system Xc− or GPX4 inhibitors represents another promising preclinical approach (148). Combining metabolic interventions with immune checkpoint blockade may be particularly attractive because metabolic reprogramming could simultaneously increase tumour-cell vulnerability and restore immune-cell function.

Engineered extracellular vesicles may provide a means of delivering metabolic inhibitors, siRNAs or immune modulators directly to the tumour (149). Their ability to cross biological barriers makes them attractive, although delivery efficiency, cargo heterogeneity and manufacturing remain important translational challenges.

### 6.3 Combined immune checkpoint strategies

The limited efficacy of PD-1/PD-L1 blockade in GBM is now documented by three negative phase III trials: CheckMate-143 in recurrent disease (median OS 9.8 vs 10.0 months for nivolumab vs bevacizumab; HR 1.04; ORR 7.8% vs 23.1%), CheckMate-498 in newly diagnosed MGMT-unmethylated disease (median OS 13.4 vs 14.9 months, favouring temozolomide) and CheckMate-548 in newly diagnosed MGMT-methylated disease (no PFS or OS benefit) (150–152). This failure reflects GBM-specific biological barriers rather than insensitivity of the PD-1/PD-L1 axis itself. First, GBM is an immunologically ‘cold’ tumour with sparse effector T-cell infiltration, a low tumour mutational burden and scarce neoantigens, limiting the pool of reactivatable T cells. Second, its TIME is dominated by immunosuppressive myeloid cells—TAMs and MDSCs may constitute 30–50% of the tumour mass (47)—which sustain suppression even when PD-1 signalling is blocked. Third, the blood-brain and blood–tumour barriers restrict both antibody penetration and immune-cell trafficking, whilst bone-marrow sequestration of T cells and treatment-associated lymphopenia (corticosteroids, chemoradiation) further deplete circulating effectors (102). Fourth, compensatory checkpoints (TIM-3, LAG-3, TIGIT and emerging molecules such as BTN2A2) and epigenetically fixed, TOX-driven T-cell exhaustion render single-checkpoint relief insufficient (100, 101, 139). Combination strategies therefore aim to simultaneously relieve T-cell inhibition and remodel the myeloid-dominated TIME.

Combining PD-1/PD-L1 blockade with TGF-β inhibition, CSF-1R inhibition or CXCR2 blockade could theoretically reduce Treg and myeloid suppression whilst enhancing T-cell infiltration (53, 78). However, these combinations remain under active investigation, and clinical benefit should not be inferred from preclinical synergy.

CD47/SIRPα blockade aims to restore macrophage phagocytosis (80). Although magrolimab (Hu5F9-G4) advanced in haematological malignancies, its development in several settings was limited by efficacy and safety concerns, emphasising the need for improved dosing and next-generation CD47/SIRPα agents. Early-phase investigation in brain tumours continues, including PNOC025 (NCT05169944) (153). Haematological toxicity remains an important concern because CD47 is expressed on normal blood cells.

FcγRIIB has also emerged as a potential modulator of antibody and checkpoint responses (154). Bispecific or combination approaches targeting Fc receptor pathways together with PD-L1 may enhance anti-tumour immunity, although this concept remains experimental.

Other approaches include dual-checkpoint or bispecific antibodies targeting PD-L1 together with CTLA-4, TGF-β or other inhibitory pathways. The rationale is to overcome the multiple layers of GSC- and TIME-mediated immune suppression rather than relying on a single checkpoint.

### 6.4 Cell therapy

EGFRvIII-specific CAR-T therapy provided an important proof of concept for adoptive cellular therapy in GBM. Although early studies demonstrated feasibility and safety, clinical responses have generally been transient, potentially reflecting antigen heterogeneity, antigen loss and TIME-mediated suppression (155). Subsequent trials have reproduced this pattern: locoregional IL-13Rα2-targeting CAR-T cells showed bioactivity in a phase 1 trial of recurrent high-grade glioma, although responses were largely transient (156); intraventricular EGFRvIII-directed CARv3-TEAM-E T cells induced rapid radiographic regression within days in the first three treated patients, but responses were short-lived (157); and intrathecal bivalent EGFR × IL-13Rα2 CAR-T cells showed phase 1 feasibility and early bioactivity, with reduced contrast enhancement but no objective radiographic responses. Testing of this bivalent construct is continuing with the aim of improving antigen coverage and reducing antigen escape (158).

CD44 and CD133 represent additional GSC-associated targets, and dual-targeting IL-7Rα-armoured CAR-T cells against these antigens have shown potent preclinical efficacy (159). Such dual-target CAR-T approaches may improve tumour coverage compared with single-antigen targeting, although antigen heterogeneity and on-target/off-tumour toxicity remain concerns.

NK-cell therapy offers complementary advantages because NK cells can recognise malignant cells through multiple activating and inhibitory receptors. Engineering approaches include TGF-β receptor disruption and enhanced activating-receptor signalling (160). CAR-NK cells are also being investigated as potentially safer alternatives to CAR-T therapy.

Cytokine-armoured cellular therapies, including IL-15-modified CAR-T cells, may improve persistence within the immunosuppressive GBM environment (161). Other approaches use mesenchymal stromal cells or engineered delivery systems to simultaneously modulate GSC stemness and immune checkpoints (162). Targeting MDSCs through engineered immune cells represents another potential strategy (163).

CRISPR-based functional screens have further identified genes involved in GSC adaptation to CAR-T pressure, providing potential targets for overcoming cellular-therapy resistance (10). Nevertheless, antigen heterogeneity, T-cell exhaustion, poor trafficking, the blood-brain/tumour barriers and TIME-mediated suppression remain major obstacles (102, 164).

### 6.5 Other emerging strategies

Epigenetic therapy may reverse the stemness-associated state of GSCs. HDAC inhibitors such as vorinostat and panobinostat can promote differentiation and may enhance sensitivity to radiotherapy or chemotherapy (47, 165). EZH2 and G9a inhibition similarly target epigenetic programmes supporting GSC maintenance (24, 166).

Notch signalling represents another potential target. γ-secretase inhibitors such as RO4929097 and MK0752 have been evaluated clinically but showed limited single-agent activity (167). Combination approaches targeting Notch signalling together with radiotherapy or immune modulation may therefore be more promising.

Extracellular-vesicle biology provides another opportunity to disrupt GSC–TIME communication. Inhibiting exosome biogenesis or release with agents such as GW4869 may reduce the transfer of immunosuppressive cargo (168, 169). Conversely, engineered or interferon-conditioned extracellular vesicles could potentially compete with tumour-derived vesicles or deliver therapeutic cargo (170).

Finally, AAV-mediated LIGHT expression represents a novel strategy to convert the GBM microenvironment into a more immune-permissive state. AAV-LIGHT can promote high endothelial venule formation and TLS development, thereby enhancing immune-cell recruitment and potentially converting an immunologically “cold” tumour into a more inflamed environment (171). These approaches remain experimental and require rigorous assessment of delivery, durability and safety.

Despite this expanding therapeutic repertoire, clinical translation faces modality-specific barriers that should be considered when prioritising strategies. Small-molecule inhibitors of metabolic and signalling nodes (for example, LDHA, glutaminase, γ-secretase and CSF-1R inhibitors) are frequently constrained by limited blood-brain barrier penetration and by efflux through ABC transporters such as P-glycoprotein and BCRP, and few have demonstrated adequate intratumoural exposure in patients. Antibody-based agents face distinct limitations: CD47/SIRPα blockade is complicated by on-target haematological toxicity—anaemia and thrombocytopenia—because CD47 is ubiquitously expressed on erythrocytes and platelets (153), whilst the size of full-length antibodies and bispecific constructs restricts their central nervous system bioavailability. Cell-based therapies must additionally overcome inefficient trafficking across the blood–tumour barrier, antigen heterogeneity and antigen loss, and the logistical complexity of manufacturing (102, 164). Extracellular vesicle-based delivery remains limited by cargo heterogeneity, unfavourable biodistribution and the lack of scalable, GMP-compliant production (149). These modality-specific hurdles argue for rational combination design, delivery innovations (including focused ultrasound, convection-enhanced delivery and locoregional administration) and biomarker-guided patient selection as prerequisites for successful clinical translation. Collectively, these therapeutic modalities target distinct nodes of GSC–TIME crosstalk through small-molecule inhibition, antibody-based checkpoint modulation, cell therapy and engineered delivery systems. Despite their different mechanisms, they face shared barriers, including BBB/CNS delivery, antigen escape, myeloid suppression and systemic toxicity. Rational combination design, delivery innovation and biomarker-guided patient selection are therefore required for clinical translation (**Figure 3**).

**Figure 3:**
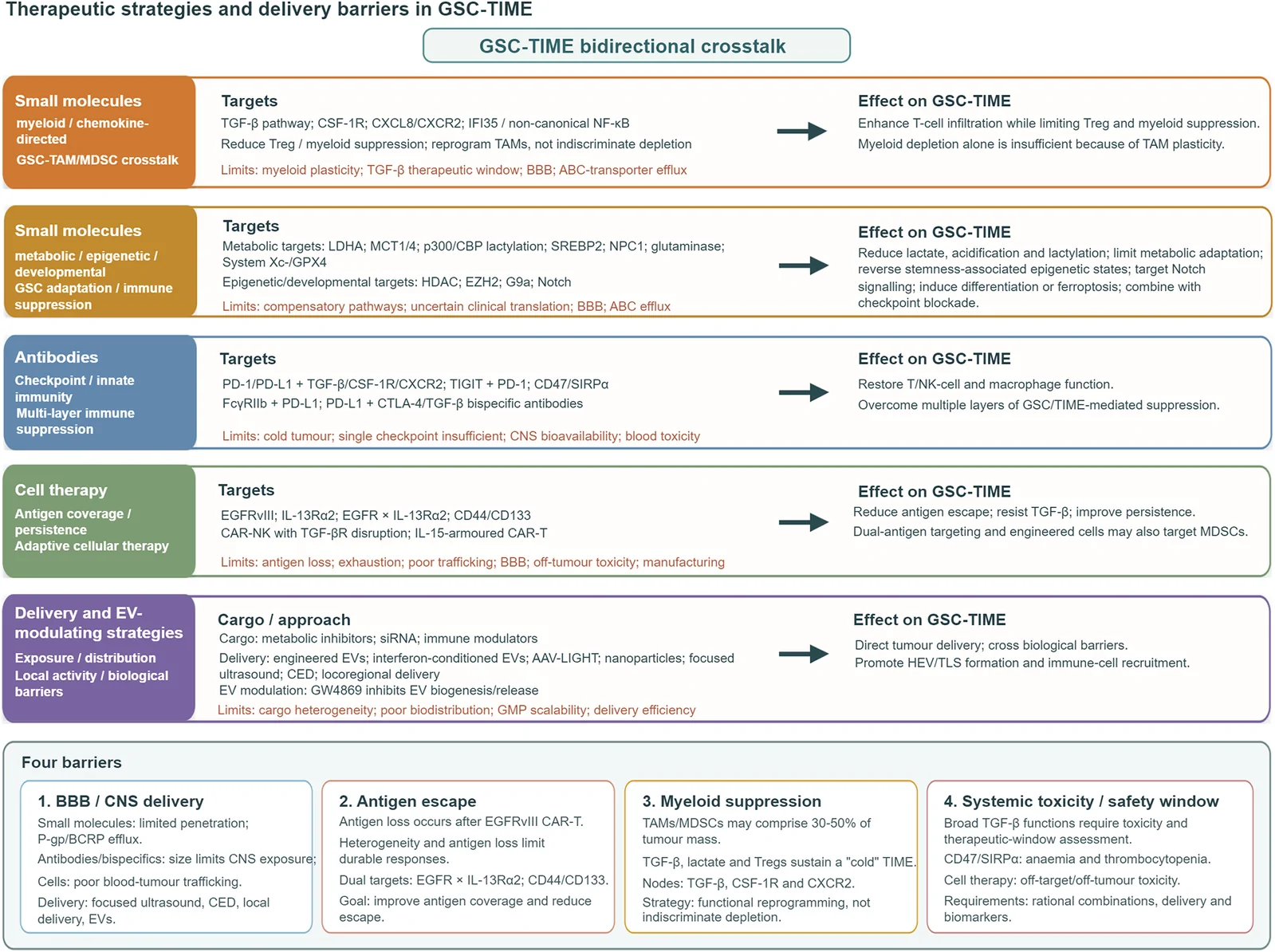
Therapeutic strategies and delivery barriers in GSC–TIME crosstalk. The schematic summarises five therapeutic approaches, their principal targets, intended effects and translational limitations. Small-molecule strategies are divided into myeloid/chemokine-directed agents targeting TGF-β, CSF-1R, IL-8/CXCR2 and IFI35/non-canonical NF-κB signalling, and metabolic, epigenetic or developmental agents targeting LDHA, MCT1/4, lactylation, SREBP2, NPC1, glutaminase, system Xc−/GPX4, HDAC, EZH2, G9a and Notch. Antibody-based approaches include PD-1/PD-L1 combinations, TIGIT plus PD-1, CD47/SIRPα, FcγRIIB plus PD-L1 and PD-L1-directed bispecific antibodies. Cell therapies target EGFRvIII, IL-13Rα2, EGFR × IL-13Rα2 and CD44/CD133, with additional engineering to disrupt TGF-β signalling or enhance persistence. Delivery and EV-modulating strategies include engineered extracellular vesicles, interferon-conditioned EVs, GW4869, AAV-LIGHT, nanoparticles, focused ultrasound, convection-enhanced delivery (CED) and locoregional administration. The bottom panels highlight four barriers: blood-brain barrier (BBB)/blood-tumour barrier and CNS delivery, antigen escape, myeloid suppression and systemic toxicity/safety window. The image also indicates that most mechanisms remain primarily preclinical and that clinical benefit cannot be inferred directly from preclinical synergy.

## 7 Conclusions and prospects

GSC–TIME crosstalk constitutes a dynamic and bidirectional regulatory system in which GSCs actively construct an immunosuppressive niche, whilst immune and stromal cells reciprocally reinforce GSC stemness, survival and treatment resistance. This reciprocal interaction provides an important mechanistic framework for understanding GBM recurrence and the limited efficacy of current immunotherapies.

Several signalling networks, including B2M/TGF-β1, IL-8/CXCR2, IFI35/NF-κB and lactate/lactylation, illustrate how cytokine signalling, chemotaxis and tumour metabolism converge on GSC–myeloid interactions. Emerging mechanisms involving IgG/FcγRIIA–AKT/mTOR, WTAP/m6A/LOXL2 and PTEN-associated metabolic modification further demonstrate the increasing complexity of this network. However, the strength of evidence underlying these pathways varies considerably: most remain validated primarily in preclinical systems, and only a minority are corroborated by patient-derived single-cell, spatial or clinical-trial data. Prioritising mechanisms with direct human evidence may therefore improve the likelihood of successful translation.

Future therapeutic development should therefore focus on rational combination strategies rather than single-node inhibition. Three priorities are particularly compelling: first, simultaneous targeting of metabolic and epigenetic vulnerabilities; second, next-generation cellular therapies designed to resist TGF-β, metabolic stress and antigen escape; and third, spatial reprogramming of the TIME through selective modulation of TAMs, MDSCs, Tregs and TLS formation. Strategies targeting the blood-brain and blood–tumour barriers, including focused ultrasound, nanoparticles and engineered extracellular vesicles, may further improve delivery of these interventions.

Importantly, future studies should incorporate single-cell and spatial multi-omics, longitudinal sampling and clinically relevant models to determine which GSC–TIME interactions are dominant in individual patients and at specific stages of treatment. Such an approach may enable biomarker-guided combination therapy rather than empiric multi-agent treatment.

In summary, understanding GSC–TIME crosstalk shifts the therapeutic focus from targeting tumour cells or immune cells in isolation towards disrupting the reciprocal ecosystem that sustains GBM. Precisely identifying and therapeutically intercepting these context-dependent interactions may provide a more effective route towards preventing immune escape, treatment resistance and tumour recurrence.

## Glossary

AAV: adeno-associated virus
ARG1: arginase 1
B2M: β2-microglobulin
Bregs: regulatory B cells
CAR-T: chimeric antigen receptor T cell
CSF-1R: colony-stimulating factor 1 receptor
CTLA-4: cytotoxic T-lymphocyte-associated protein 4
EGFRvIII: epidermal growth factor receptor (variant III)
EV: extracellular vesicle
GBM: glioblastoma
GSC: glioblastoma stem cell
HDAC: histone deacetylase
HIF: hypoxia-inducible factor
IDO: indoleamine 2,3-dioxygenase
IFI35: interferon alpha inducible protein 35
iNOS: inducible nitric oxide synthase
LDHA: lactate dehydrogenase A
m^6^A: N^6^-methyladenosine
MDSC: myeloid-derived suppressor cell
E-MDSC: early-stage MDSC
M-MDSC: monocytic MDSC
MGMT: O^6^-methylguanine-DNA methyltransferase
MMP: matrix metalloproteinase
NET: neutrophil extracellular trap
NF-κB: nuclear factor-κB
NK: natural killer
PD-1: programmed death-1
PD-L1: programmed death ligand-1
PMN-MDSC: polymorphonuclear MDSC
scRNA-seq: single-cell RNA sequencing
SHH: sonic hedgehog
SIRPα: signal regulatory protein α
SREBP2: sterol regulatory element-binding protein 2
TAA: tumour-associated astrocyte
TAM: tumour-associated macrophage/microglia
TAN: tumour-associated neutrophil
TGF-β: transforming growth factor-β
TIME: tumour immune microenvironment
TLS: tertiary lymphoid structure
TMZ: temozolomide
Treg: regulatory T cell
TREM2: triggering receptor expressed on myeloid cells 2
VEGF: vascular endothelial growth factor

## References

[1] Price M Ballard CAP Benedetti JR Kruchko C Barnholtz-Sloan JS Ostrom QT . Cbtrus statistical report: Primary brain and other central nervous system tumors diagnosed in the United States in 2018-2022. Neuro Oncol. (2025) 27:iv1–iv66. doi: 10.1093/neuonc/noaf194 41092086 PMC12527013

[2] Singh S Dey D Barik D Mohapatra I Kim S Sharma M . Glioblastoma at the crossroads: Current understanding and future therapeutic horizons. Signal Transduct Target Ther. (2025) 10:213. doi: 10.1038/s41392-025-02299-4 40628732 PMC12238593

[3] Stupp R Mason WP van den Bent MJ Weller M Fisher B Taphoorn MJ . Radiotherapy plus concomitant and adjuvant temozolomide for glioblastoma. N Engl J Med. (2005) 352:987–96. doi: 10.1056/NEJMoa043330 15758009

[4] Chinot OL Wick W Mason W Henriksson R Saran F Nishikawa R . Bevacizumab plus radiotherapy-temozolomide for newly diagnosed glioblastoma. N Engl J Med. (2014) 370:709–22. doi: 10.1056/NEJMoa1308345 24552318

[5] Gimple RC Bhargava S Dixit D Rich JN . Glioblastoma stem cells: Lessons from the tumor hierarchy in a lethal cancer. Genes Dev. (2019) 33:591–609. doi: 10.1101/gad.324301.119 31160393 PMC6546059

[6] Yang J Li J Jin M Lu Y Zhang L . The immunosuppressive tumor microenvironment in glioblastoma. Front Immunol. (2026) 17:1806996. doi: 10.3389/fimmu.2026.1806996 42465775 PMC13373089

[7] Li J Ross JL Hambardzumyan D Brat DJ . Immunopathology of glioblastoma. Annu Rev Pathol. (2026) 21:135–62. doi: 10.1146/annurev-pathmechdis-042524-025950 41061166

[8] Kirschenbaum D Xie K Ingelfinger F Katzenelenbogen Y Abadie K Look T . Time-resolved single-cell transcriptomics defines immune trajectories in glioblastoma. Cell. (2024) 187:149–65.e23. doi: 10.1016/j.cell.2023.11.032 38134933

[9] Wang X Sun Q Wang W Liu B Gu Y Chen L . Decoding key cell sub-populations and molecular alterations in glioblastoma at recurrence by single-cell analysis. Acta Neuropathol Commun. (2023) 11:125. doi: 10.1186/s40478-023-01613-x 37525259 PMC10391841

[10] Wang D Prager BC Gimple RC Aguilar B Alizadeh D Tang H . Crispr screening of car t cells and cancer stem cells reveals critical dependencies for cell-based therapies. Cancer Discov. (2021) 11:1192–211. doi: 10.1158/2159-8290.CD-20-1243 33328215 PMC8406797

[11] Vora P Venugopal C McFarlane N Singh SK . Culture and isolation of brain tumor initiating cells. Curr Protoc Stem Cell Biol. (2015) 34:3 1–3 13. doi: 10.1002/9780470151808.sc0303s34 26237571

[12] Galli R Binda E Orfanelli U Cipelletti B Gritti A De Vitis S . Isolation and characterization of tumorigenic, stem-like neural precursors from human glioblastoma. Cancer Res. (2004) 64:7011–21. doi: 10.1158/0008-5472.CAN-04-1364 15466194

[13] Vora P Venugopal C Salim SK Tatari N Bakhshinyan D Singh M . The rational development of cd133-targeting immunotherapies for glioblastoma. Cell Stem Cell. (2020) 26:832–44.e6. doi: 10.1016/j.stem.2020.04.008 32464096

[14] Altaner C . Glioblastoma and stem cells. Neoplasma. (2008) 55:369–74. 18665745

[15] Suva ML Rheinbay E Gillespie SM Patel AP Wakimoto H Rabkin SD . Reconstructing and reprogramming the tumor-propagating potential of glioblastoma stem-like cells. Cell. (2014) 157:580–94. doi: 10.1016/j.cell.2014.02.030 24726434 PMC4004670

[16] Kupp R Shtayer L Tien AC Szeto E Sanai N Rowitch DH . Lineage-restricted olig2-rtk signaling governs the molecular subtype of glioma stem-like cells. Cell Rep. (2016) 16:2838–45. doi: 10.1016/j.celrep.2016.08.040 27626655 PMC5024710

[17] Yang C Tian G Dajac M Doty A Wang S Lee JH . Slow-cycling cells in glioblastoma: A specific population in the cellular mosaic of cancer stem cells. Cancers (Basel). (2022) 14. doi: 10.3390/cancers14051126 35267434 PMC8909138

[18] Verhaak RG Hoadley KA Purdom E Wang V Qi Y Wilkerson MD . Integrated genomic analysis identifies clinically relevant subtypes of glioblastoma characterized by abnormalities in pdgfra, idh1, egfr, and nf1. Cancer Cell. (2010) 17:98–110. doi: 10.1016/j.ccr.2009.12.020 20129251 PMC2818769

[19] Chelebian E Fuster-Garcia E Alvarez-Torres MDM Juan-Albarracin J Garcia-Gomez JM . Higher vascularity at infiltrated peripheral edema differentiates proneural glioblastoma subtype. PloS One. (2020) 15:e0232500. doi: 10.1371/journal.pone.0232500 33052913 PMC7556526

[20] Zhang H Wu X Liao H Zhang Y . Immune niche-associated and niche-regulated plasticity in glioblastoma: State transitions, immune escape, and therapeutic vulnerabilities. Front Immunol. (2026) 17:1857659. doi: 10.3389/fimmu.2026.1857659 42433345 PMC13350452

[21] Prager BC Bhargava S Mahadev V Hubert CG Rich JN . Glioblastoma stem cells: Driving resilience through chaos. Trends Cancer. (2020) 6:223–35. doi: 10.1016/j.trecan.2020.01.009 32101725 PMC8779821

[22] Tang J Amin MA Campian JL . Glioblastoma stem cells at the nexus of tumor heterogeneity, immune evasion, and therapeutic resistance. Cells. (2025) 14. doi: 10.3390/cells14080562 40277888 PMC12025403

[23] Cao Y Liu B Cai L Li Y Huang Y Zhou Y . G9a promotes immune suppression by targeting the fbxw7/notch pathway in glioma stem cells. CNS Neurosci Ther. (2023) 29:2508–21. doi: 10.1111/cns.14191 36971192 PMC10401078

[24] Kundu S Nandhu MS Longo SL Longo JA Rai S Chin LS . The scaffolding protein dlg5 promotes glioblastoma growth by controlling sonic hedgehog signaling in tumor stem cells. Neuro Oncol. (2022) 24:1230–42. doi: 10.1093/neuonc/noac001 34984467 PMC9340653

[25] Li D Wang X Chen K Shan D Cui G Yuan W . Ifi35 regulates non-canonical nf-kappab signaling to maintain glioblastoma stem cells and recruit tumor-associated macrophages. Cell Death Differ. (2024) 31:738–52. doi: 10.1038/s41418-024-01292-8 38594444 PMC11165006

[26] Lu C Kang T Zhang J Yang K Liu Y Song K . Combined targeting of glioblastoma stem cells of different cellular states disrupts malignant progression. Nat Commun. (2025) 16:2974. doi: 10.1038/s41467-025-58366-5 40140646 PMC11947120

[27] Schonrock A Heinzelmann E Steffl B Demirdizen E Narayanan A Krunic D . Meox2 homeobox gene promotes growth of malignant gliomas. Neuro Oncol. (2022) 24:1911–24. doi: 10.1093/neuonc/noac110 35468210 PMC9629421

[28] Li H Song C Zhang Y Liu G Mi H Li Y . Transgelin promotes glioblastoma stem cell hypoxic responses and maintenance through p53 acetylation. Adv Sci (Weinh). (2024) 11:e2305620. doi: 10.1002/advs.202305620 38087889 PMC10870072

[29] Lee EJ Rath P Liu J Ryu D Pei L Noonepalle SK . Identification of global dna methylation signatures in glioblastoma-derived cancer stem cells. J Genet Genomics. (2015) 42:355–71. doi: 10.1016/j.jgg.2015.06.003 26233891 PMC4648292

[30] Hall AW Battenhouse AM Shivram H Morris AR Cowperthwaite MC Shpak M . Bivalent chromatin domains in glioblastoma reveal a subtype-specific signature of glioma stem cells. Cancer Res. (2018) 78:2463–74. doi: 10.1158/0008-5472.CAN-17-1724 29549165 PMC5955797

[31] Xie Y Li Y Tang M Li Z Hu W Qiu X . The braf v600e/mek/erk/mett3 positive feedback loop regulates autophagy and promotes stemness and invasiveness in glioblastoma via m(6)a modification. J Exp Clin Cancer Res. (2025) 45:28. doi: 10.1186/s13046-025-03623-0 41469702 PMC12853985

[32] Gu D Zhou F You H Gao J Kang T Dixit D . Sterol regulatory element-binding protein 2 maintains glioblastoma stem cells by keeping the balance between cholesterol biosynthesis and uptake. Neuro Oncol. (2023) 25:1578–91. doi: 10.1093/neuonc/noad060 36934350 PMC10651206

[33] Chung C Sweha SR Pratt D Tamrazi B Panwalkar P Banda A . Integrated metabolic and epigenomic reprograming by h3k27m mutations in diffuse intrinsic pontine gliomas. Cancer Cell. (2020) 38:334–49.e9. doi: 10.1016/j.ccell.2020.07.008 32795401 PMC7494613

[34] Khaket TP Rimal S Chavda V Lu B . Mitochondrial reverse electron transport regulates glioma stemness through sirt3-hif1alpha-sox2 signaling. Cancer Lett. (2026) 645:218354. doi: 10.1016/j.canlet.2026.218354 41740830

[35] Yin J Ge X Ding F He L Song K Shi Z . Reactivating pten to impair glioma stem cells by inhibiting cytosolic iron-sulfur assembly. Sci Transl Med. (2024) 16:eadg5553. doi: 10.1126/scitranslmed.adg5553 38507470

[36] Wang P Gong S Liao B Pan J Wang J Zou D . Hif1alpha/hif2alpha induces glioma cell dedifferentiation into cancer stem cells through sox2 under hypoxic conditions. J Cancer. (2022) 13:1–14. doi: 10.7150/jca.54402 34976166 PMC8692689

[37] Wu X Yuan H Wu Q Gao Y Duan T Yang K . Threonine fuels glioblastoma through yrdc-mediated codon-biased translational reprogramming. Nat Cancer. (2024) 5:1024–44. doi: 10.1038/s43018-024-00748-7 38519786 PMC11552442

[38] Gabrusiewicz K Li X Wei J Hashimoto Y Marisetty AL Ott M . Glioblastoma stem cell-derived exosomes induce m2 macrophages and pd-l1 expression on human monocytes. Oncoimmunology. (2018) 7:e1412909. doi: 10.1080/2162402X.2017.1412909 29632728 PMC5889290

[39] Domenis R Cesselli D Toffoletto B Bourkoula E Caponnetto F Manini I . Systemic t cells immunosuppression of glioma stem cell-derived exosomes is mediated by monocytic myeloid-derived suppressor cells. PloS One. (2017) 12:e0169932. doi: 10.1371/journal.pone.0169932 28107450 PMC5249124

[40] Fan Y Gao Z Xu J Wang H Guo Q Li B . Spi1-mediated mir222hg transcription promotes proneural-to-mesenchymal transition of glioma stem cells and immunosuppressive polarization of macrophages. Theranostics. (2023) 13:3310–29. doi: 10.7150/thno.82590 37351164 PMC10283056

[41] Yang J Sun G Hu Y Yang J Shi Y Liu H . Extracellular vesicle lncrna metastasis-associated lung adenocarcinoma transcript 1 released from glioma stem cells modulates the inflammatory response of microglia after lipopolysaccharide stimulation through regulating mir-129-5p/high mobility group box-1 protein axis. Front Immunol. (2019) 10:3161. doi: 10.3389/fimmu.2019.03161 32117213 PMC7020807

[42] Spinelli C Adnani L Meehan B Montermini L Huang S Kim M . Mesenchymal glioma stem cells trigger vasectasia-distinct neovascularization process stimulated by extracellular vesicles carrying egfr. Nat Commun. (2024) 15:2865. doi: 10.1038/s41467-024-46597-x 38570528 PMC10991552

[43] Sun X Ma X Wang J Zhao Y Wang Y Bihl JC . Glioma stem cells-derived exosomes promote the angiogenic ability of endothelial cells through mir-21/vegf signal. Oncotarget. (2017) 8:36137–48. doi: 10.18632/oncotarget.16661 28410224 PMC5482644

[44] Ai Y Zhou J . Mechanotransduction in glioma stem cell fate determination: From niche mechanics to therapeutic vulnerability and state plasticity. Front Cell Dev Biol. (2026) 14:1849535. doi: 10.3389/fcell.2026.1849535 42368454 PMC13303627

[45] Yan K Wu Q Yan DH Lee CH Rahim N Tritschler I . Glioma cancer stem cells secrete gremlin1 to promote their maintenance within the tumor hierarchy. Genes Dev. (2014) 28:1085–100. doi: 10.1101/gad.235515.113 24788093 PMC4035537

[46] Kelly JJ Stechishin O Chojnacki A Lun X Sun B Senger DL . Proliferation of human glioblastoma stem cells occurs independently of exogenous mitogens. Stem Cells. (2009) 27:1722–33. doi: 10.1002/stem.98 19544433

[47] Cheng Y Zhao W Xie M Zheng X Ding F Du J . Decoding myeloid heterogeneity in glioblastoma: Spatial insights from transcriptomics. J Transl Med. (2026) 24. doi: 10.1186/s12967-026-07873-0 41723505 PMC13032637

[48] Zhang L Jiang Y Zhang G Wei S . The diversity and dynamics of tumor-associated macrophages in recurrent glioblastoma. Front Immunol. (2023) 14:1238233. doi: 10.3389/fimmu.2023.1238233 37731483 PMC10507272

[49] Zhang X Jiang M Niu W Xu B Zhang C Yang M . Circprkd3-loaded exosomes concomitantly elicit tumor growth inhibition and glioblastoma microenvironment remodeling via inhibiting stat3 signaling. Neuro Oncol. (2025) 27:1987–2005. doi: 10.1093/neuonc/noaf019 39868921 PMC12448806

[50] Zhong J Xing X Gao Y Pei L Lu C Sun H . Distinct roles of trem2 in central nervous system cancers and peripheral cancers. Cancer Cell. (2024) 42:968–84.e9. doi: 10.1016/j.ccell.2024.05.001 38788719

[51] Bronte V Brandau S Chen SH Colombo MP Frey AB Greten TF . Recommendations for myeloid-derived suppressor cell nomenclature and characterization standards. Nat Commun. (2016) 7:12150. doi: 10.1038/ncomms12150 27381735 PMC4935811

[52] Da W Dai Y Shen B Zhang Y Bao P Zhu W . Histone lactylation-derived Tet2 enhanced Arg1-mediated Mdsc immunosuppression. Front Immunol. (2025) 16:1677780. doi: 10.3389/fimmu.2025.1677780 41624845 PMC12855476

[53] Rodriguez PC Quiceno DG Zabaleta J Ortiz B Zea AH Piazuelo MB . Arginase I production in the tumor microenvironment by mature myeloid cells inhibits T-cell receptor expression and antigen-specific T-cell responses. Cancer Res. (2004) 64:5839–49. doi: 10.1158/0008-5472.CAN-04-0465 15313928

[54] Jackson C Cherry C Bom S Dykema AG Wang R Thompson E . Distinct myeloid-derived suppressor cell populations in human glioblastoma. Science. (2025) 387:eabm5214. doi: 10.1126/science.abm5214 39818911 PMC12836367

[55] Jung MY Aibaidula A Brown DA Himes BT Cumba Garcia LM Parney IF . Superinduction of immunosuppressive glioblastoma extracellular vesicles by Ifn-gamma through Pd-L1 and Ido1. Neuro-Oncol Adv. (2022) 4:vdac017. doi: 10.1093/noajnl/vdac017 35990703 PMC9389426

[56] Himes BT Peterson TE de Mooij T Garcia LMC Jung MY Uhm S . The role of extracellular vesicles and Pd-L1 in glioblastoma-mediated immunosuppressive monocyte induction. Neuro Oncol. (2020) 22:967–78. doi: 10.1093/neuonc/noaa029 32080744 PMC7339906

[57] Lim M Ye X Piotrowski AF Desai A Ahluwalia MS Walbert T . Anti-Lag-3 with or without anti-Pd-1 in recurrent glioblastoma: A phase 1 trial. Nat Med. (2026) 32:2449–60. doi: 10.1038/s41591-026-04475-7 42432293 PMC13375532

[58] Li J Liu Y Peng P Hu J . Targeting regulatory T cells in glioblastoma: From mechanistic insights to novel immunotherapeutic strategies. Front Immunol. (2026) 17:1759023. doi: 10.3389/fimmu.2026.1759023 42039205 PMC13106435

[59] Zhang R Ma P Tang K Cao Y Yang Y Hao S . Natural killer cell dysfunction in glioma: From immune evasion to immunotherapy. Front Immunol. (2026) 17:1787023. doi: 10.3389/fimmu.2026.1787023 41929521 PMC13038952

[60] Wang Z Wu H Tan X Chen Z Chang C Zhang D . A novel plasma cell infiltration-associated prognostic signature suggests a potential role for Cabp4 in glioma progression. Brain Res. (2026) 1880:150231. doi: 10.1016/j.brainres.2026.150231 41747848

[61] Li F Liu A Zhao M Luo L . Astrocytic chitinase-3-like protein 1 in neurological diseases: Potential roles and future perspectives. J Neurochem. (2023) 165:772–90. doi: 10.1111/jnc.15824 37026513

[62] Henrik Heiland D Ravi VM Behringer SP Frenking JH Wurm J Joseph K . Tumor-associated reactive astrocytes aid the evolution of immunosuppressive environment in glioblastoma. Nat Commun. (2019) 10:2541. doi: 10.1038/s41467-019-10493-6 31186414 PMC6559986

[63] Chen Z Yuan SJ Li K Zhang Q Li TF An HC . Doxorubicin-polyglycerol-nanodiamond conjugates disrupt Stat3/Il-6-mediated reciprocal activation loop between glioblastoma cells and astrocytes. J Control Release. (2020) 320:469–83. doi: 10.1016/j.jconrel.2020.01.044 31987922

[64] Sun W Chen P Xu XY Zhang JQ Jin WL . Astrocytes in neuroinflammation and brain cancer. Mol BioMed. (2026) 7. doi: 10.1186/s43556-026-00439-y 41910655 PMC13035979

[65] Mikolajczak K Chmiel J Leszek J . Neuroinflammation in Alzheimer's disease (Ad) and glioblastoma (Gbm): Shared mechanisms and therapeutic insights. Cells. (2026) 15. doi: 10.3390/cells15121111 42346137 PMC13296939

[66] Kim DH Park HJ Lim S Koo JH Lee HG Choi JO . Regulation of chitinase-3-like-1 in T cell elicits Th1 and cytotoxic responses to inhibit lung metastasis. Nat Commun. (2018) 9:503. doi: 10.1038/s41467-017-02731-6 29403003 PMC5799380

[67] Chen A Jiang Y Li Z Wu L Santiago U Zou H . Chitinase-3-like 1 protein complexes modulate macrophage-mediated immune suppression in glioblastoma. J Clin Invest. (2021) 131. doi: 10.1172/JCI147552 34228644 PMC8363281

[68] Sun X Wang X Zhang S Long Y Wang D Gou C . Oligonucleotide G3t19 downregulates Cd44 succinylation and inhibits astrocyte-induced glioma immune escape. Cell Commun Signal. (2026). doi: 10.1186/s12964-026-03081-x 42482053 PMC13670475

[69] Tralongo P Ballato M Zuccala V Fiorentino V Giordano W Casili G . Neurotransmitter-mediated signaling in glioblastoma and glial tumors: Biology and therapeutic opportunities. Oncol Res. (2026) 34:1. doi: 10.32604/or.2026.076088 42220436 PMC13220060

[70] Rong Y Durden DL Van Meir EG Brat DJ . 'Pseudopalisading' necrosis in glioblastoma: A familiar morphologic feature that links vascular pathology, hypoxia, and angiogenesis. J Neuropathol Exp Neurol. (2006) 65:529–39. doi: 10.1097/00005072-200606000-00001 16783163

[71] Biserova K Strumfa I . Glioblastoma stem cells and tumour microenvironment: Interactions across hypoxia, vasculature and immune modulation. Int J Mol Sci. (2026) 27. doi: 10.3390/ijms27062557 41898421 PMC13027279

[72] Yuan Y Liu X Kuang L Yang S Wang L Wang J . Endothelial cell-derived Sdf-1alpha elicits stemness traits of glioblastoma via dual-regulation of Gli1. Theranostics. (2025) 15:9819–37. doi: 10.7150/thno.108843 41041071 PMC12486407

[73] Zheng ZQ Chen JT Zheng MC Yang LJ Wang JM Liu QL . Nestin+/Cd31+ cells in the hypoxic perivascular niche regulate glioblastoma chemoresistance by upregulating Jag1 and Dll4. Neuro Oncol. (2021) 23:905–19. doi: 10.1093/neuonc/noaa265 33249476 PMC8168822

[74] Cakmak P Lun JH Singh A Macas J Schupp J Schuck J . Spatial immune profiling defines a subset of human gliomas with functional tertiary lymphoid structures. Immunity. (2025) 58:2847–63.e8. doi: 10.1016/j.immuni.2025.09.018 41125076

[75] Prakash P Trippett J Ehsan C Namkung J Lad M Aghi MK . Tumor microenvironment shapes the spatial organization of glioblastoma cell states. Neuro Oncol. (2026) 28:585–96. doi: 10.1093/neuonc/noag003 41520155 PMC13070500

[76] Herisson F Frodermann V Courties G Rohde D Sun Y Vandoorne K . Direct vascular channels connect skull bone marrow and the brain surface enabling myeloid cell migration. Nat Neurosci. (2018) 21:1209–17. doi: 10.1038/s41593-018-0213-2 30150661 PMC6148759

[77] Li D Zhang Q Li L Chen K Yang J Dixit D . Beta2-microglobulin maintains glioblastoma stem cells and induces M2-like polarization of tumor-associated macrophages. Cancer Res. (2022) 82:3321–34. doi: 10.1158/0008-5472.CAN-22-0507 35841593

[78] Yuan W Zhang Q Gu D Lu C Dixit D Gimple RC . Dual role of Cxcl8 in maintaining the mesenchymal state of glioblastoma stem cells and M2-like tumor-associated macrophages. Clin Cancer Res. (2023) 29:3779–92. doi: 10.1158/1078-0432.CCR-22-3273 37439870

[79] Gan C Wu D Yuan Y Li L Bai J Xu Y . Pfkfb4 promotes M2 polarization of tumor-associated macrophages through aerobic glycolysis-mediated modification of histone H3k18 lactylation in hepatocellular carcinoma. Cancer Metab. (2026) 14. doi: 10.1186/s40170-026-00431-8 41987314 PMC13182095

[80] Wang S Huang T Wu Q Yuan H Wu X Yuan F . Lactate reprograms glioblastoma immunity through Cbx3-regulated histone lactylation. J Clin Invest. (2024) 134. doi: 10.1172/JCI176851 39545414 PMC11563687

[81] Gu D Hu L Yang K Yuan W Shan D Gao J . Stress-induced pro-inflammatory glioblastoma stem cells secrete Tnfaip6 to enhance tumor growth and induce suppressive macrophages. Dev Cell. (2025) 60:2558–75.e6. doi: 10.1016/j.devcel.2025.04.027 40403724

[82] Qiu J Zhao R Ma C Wang Q Li B Qi Y . O-Glcnacylation stabilized Wtap promotes Gbm malignant progression in an N6-methyladenosine-dependent manner. Neuro Oncol. (2025) 27:900–15. doi: 10.1093/neuonc/noae268 39671515 PMC12083224

[83] Guo M Yuan Z Jin X Li X Deng Y Zhou S . Inhibition of Icam1 diminishes stemness and enhances antitumor immunity in glioblastoma via beta-catenin/Pd-L1 signaling. Nat Commun. (2025) 16:8642. doi: 10.1038/s41467-025-63796-2 41028719 PMC12484827

[84] Qiu W Guo Q Guo X Wang C Li B Qi Y . Mesenchymal stem cells, as glioma exosomal immunosuppressive signal multipliers, enhance Mdscs immunosuppressive activity through the Mir-21/Sp1/Dnmt1 positive feedback loop. J Nanobiotechnology. (2023) 21:233. doi: 10.1186/s12951-023-01997-x 37481646 PMC10362641

[85] Mao J Li J Chen J Wen Q Cao M Zhang F . Cxcl10 and Nrf2-upregulated mesenchymal stem cells reinvigorate T lymphocytes for combating glioblastoma. J Immunother Cancer. (2023) 11. doi: 10.1136/jitc-2023-007481 38056897 PMC10711923

[86] Lea ST Chen CH Wei J William I Lopez Del Castillo I Curran MA . Nlrp3 inflammasome activation expands the immunosuppressive myeloid stroma and antagonizes the therapeutic benefit of Sting activation in glioblastoma. Cancer Res Commun. (2025) 5:960–72. doi: 10.1158/2767-9764.CRC-23-0189 40377974 PMC12163576

[87] Asad M Inocencio J Mitrasinovic S Aoki M Baker CE Hormigo A . Tigit expression dictates the immunosuppressive reprogramming of myeloid cells in glioblastoma. Neuro Oncol. (2026) 28:1426–41. doi: 10.1093/neuonc/noag044 41741381 PMC13231175

[88] Takacs GP Kreiger CJ Luo D Tian G Garcia JS Deleyrolle LP . Glioma-derived Ccl2 and Ccl7 mediate migration of immune suppressive Ccr2(+)/Cx3cr1(+) M-Mdscs into the tumor microenvironment in a redundant manner. Front Immunol. (2022) 13:993444. doi: 10.3389/fimmu.2022.993444 36685592 PMC9854274

[89] Flores-Toro JA Luo D Gopinath A Sarkisian MR Campbell JJ Charo IF . Ccr2 inhibition reduces tumor myeloid cells and unmasks a checkpoint inhibitor effect to slow progression of resistant murine gliomas. Proc Natl Acad Sci USA. (2020) 117:1129–38. doi: 10.1073/pnas.1910856117 31879345 PMC6969504

[90] Li X Qin Y Gao P Wang H Liu Y Ren L . Decoding the prognosis-related S100a9(high) monocyte in glioblastoma using single-cell and spatial transcriptome sequencing. Onco Targets Ther. (2025) 18:1211–26. doi: 10.2147/OTT.S553018 41216330 PMC12596831

[91] Wu P Guo Y Xiao L Yuan J Tang C Dong J . Lilrb2-containing small extracellular vesicles from glioblastoma promote tumor progression by promoting the formation and expansion of myeloid-derived suppressor cells. Cancer Immunol Immunother. (2023) 72:2179–93. doi: 10.1007/s00262-023-03395-6 36853330 PMC10264499

[92] Tian Y Gao X Yang X Chen S Ren Y . Vegfa contributes to tumor property of glioblastoma cells by promoting differentiation of myeloid-derived suppressor cells. BMC Cancer. (2024) 24:1040. doi: 10.1186/s12885-024-12803-8 39174921 PMC11342494

[93] Guo X Qiu W Wang J Liu Q Qian M Wang S . Glioma exosomes mediate the expansion and function of myeloid-derived suppressor cells through Microrna-29a/Hbp1 and Microrna-92a/Prkar1a pathways. Int J Cancer. (2019) 144:3111–26. doi: 10.1002/ijc.32052 30536597

[94] Guo X Qiu W Liu Q Qian M Wang S Zhang Z . Immunosuppressive effects of hypoxia-induced glioma exosomes through myeloid-derived suppressor cells via the Mir-10a/Rora and Mir-21/Pten pathways. Oncogene. (2018) 37:4239–59. doi: 10.1038/s41388-018-0261-9 29713056

[95] Qiu W Guo X Li B Wang J Qi Y Chen Z . Exosomal Mir-1246 from glioma patient body fluids drives the differentiation and activation of myeloid-derived suppressor cells. Mol Ther. (2021) 29:3449–64. doi: 10.1016/j.ymthe.2021.06.023 34217892 PMC8636176

[96] Tian Y Gao X Yang X Chen S Ren Y . Glioma-derived exosome Lncrna Agap2-As1 promotes glioma proliferation and metastasis by mediating Tgf-beta1 secretion of myeloid-derived suppressor cells. Heliyon. (2024) 10:e29949. doi: 10.1016/j.heliyon.2024.e29949 38699039 PMC11064146

[97] Wang B Zhao X Han S Xu Y Li J . Crosstalk between cancer stem cells and myeloid-derived suppressor cells: Implications for tumor progression and immunotherapy. Front Immunol. (2025) 16:1691661. doi: 10.3389/fimmu.2025.1691661 41368628 PMC12682901

[98] Wang J Peng B Li T Wang T . Glioma-derived extracellular vesicles as drivers of immunotherapeutic resistance: Mechanisms of immune reprogramming and metabolic intervention. Front Immunol. (2026) 17:1859282. doi: 10.3389/fimmu.2026.1859282 42253953 PMC13236686

[99] Aydin HA Keskin E Kalayci M . Reprogramming the immunosuppressive microenvironment in glioblastoma: The microglia-Mdsc-Treg axis and translational therapeutic strategies. Front Oncol. (2026) 16:1782775. doi: 10.3389/fonc.2026.1782775 42639384 PMC13501361

[100] Dong W Li Y Wu Z Fu Q Zhao J Wang B . Cytotoxic lymphocyte heterogeneity in glioblastoma: Insights from single-cell and spatial multiomics toward precision immunotherapeutic reprogramming. Front Immunol. (2026) 17:1837084. doi: 10.3389/fimmu.2026.1837084 42358950 PMC13291131

[101] Pan X Pan Y Su Y Xu Y Du J Cheng H . Chronic Tcr signaling-driven suppression of the Foxo1-Klhl6 axis promotes T cell exhaustion. Immun Inflammation. (2026) 2:8. doi: 10.1007/s44466-025-00023-z 41541249 PMC12799747

[102] Pourmasoumi P Pourmasoumi A Zamani F Amini A Shokri S . Car-T cell therapy in glioblastoma: Overcoming immunological barriers and advancing clinical translation. Cancer Manag Res. (2026) 18:580320. doi: 10.2147/CMAR.S580320 41737136 PMC12927832

[103] Gholamin S Mitra SS Feroze AH Liu J Kahn SA Zhang M . Disrupting the Cd47-Sirpalpha anti-phagocytic axis by a humanized anti-Cd47 antibody is an efficacious treatment for malignant pediatric brain tumors. Sci Transl Med. (2017) 9. doi: 10.1126/scitranslmed.aaf2968 28298418

[104] Hung AL Maxwell R Theodros D Belcaid Z Mathios D Luksik AS . Tigit and Pd-1 dual checkpoint blockade enhances antitumor immunity and survival in Gbm. Oncoimmunology. (2018) 7:e1466769. doi: 10.1080/2162402X.2018.1466769 30221069 PMC6136875

[105] Raphael I Kumar R McCarl LH Shoger K Wang L Sandlesh P . Tigit and Pd-1 immune checkpoint pathways are associated with patient outcome and anti-tumor immunity in glioblastoma. Front Immunol. (2021) 12:637146. doi: 10.3389/fimmu.2021.637146 34025646 PMC8137816

[106] Lupo KB Matosevic S . Cd155 immunoregulation as a target for natural killer cell immunotherapy in glioblastoma. J Hematol Oncol. (2020) 13:76. doi: 10.1186/s13045-020-00913-2 32532329 PMC7291472

[107] Wu A Wei J Kong LY Wang Y Priebe W Qiao W . Glioma cancer stem cells induce immunosuppressive macrophages/microglia. Neuro Oncol. (2010) 12:1113–25. doi: 10.1093/neuonc/noq082 20667896 PMC3098021

[108] Hatiboglu MA Wei J Wu AS Heimberger AB . Immune therapeutic targeting of glioma cancer stem cells. Target Oncol. (2010) 5:217–27. doi: 10.1007/s11523-010-0151-8 20737294 PMC4262967

[109] Iwata R Hyoung Lee J Hayashi M Dianzani U Ofune K Maruyama M . Icoslg-mediated regulatory T-cell expansion and Il-10 production promote progression of glioblastoma. Neuro Oncol. (2020) 22:333–44. doi: 10.1093/neuonc/noz204 31634400 PMC7442411

[110] Liu S Zhang C Wang B Zhang H Qin G Li C . Regulatory T cells promote glioma cell stemness through Tgf-beta-Nf-kappab-Il6-Stat3 signaling. Cancer Immunol Immunother. (2021) 70:2601–16. doi: 10.1007/s00262-021-02872-0 33576874 PMC8360896

[111] Medof ME Rieder SA Shevach EM . Disabled C3ar1/C5ar1 signaling in Foxp3+ T regulatory cells leads to Tsdr demethylation and long-term stability. J Immunol. (2023) 211:1359–66. doi: 10.4049/jimmunol.2300184 37756526 PMC10591991

[112] Gao J Gu D Yang K Zhang J Lin Q Yuan W . Infiltrating plasma cells maintain glioblastoma stem cells through Igg-tumor binding. Cancer Cell. (2025) 43:122–43.e8. doi: 10.1016/j.ccell.2024.12.006 39753140

[113] Chen X Dai W Wang H Fang J Yin B Liu C . Rbfox2 suppresses netosis and glioma growth via 5hmc-dependent Pdgfb decay. Cell Rep. (2026) 45:117546. doi: 10.1016/j.celrep.2026.117546 42284147

[114] Stawicki J Wawrzyniak-Gramacka E Morawin B Grzmil A Tylutka A Zembron-Lacny A . Netosis activity in patients with glioblastoma multiforme compared to healthy controls. Sci Rep. (2026) 16. doi: 10.1038/s41598-026-51540-9 42098251 PMC13342672

[115] Wang X Li X Wu Y Hong J Zhang M . The prognostic significance of tumor-associated neutrophils and circulating neutrophils in glioblastoma (Who Cns5 classification). BMC Cancer. (2023) 23:20. doi: 10.1186/s12885-022-10492-9 36609243 PMC9817270

[116] Tao W Chu C Zhou W Huang Z Zhai K Fang X . Dual role of Wisp1 in maintaining glioma stem cells and tumor-supportive macrophages in glioblastoma. Nat Commun. (2020) 11:3015. doi: 10.1038/s41467-020-16827-z 32541784 PMC7295765

[117] Liu Z Kuang W Zhou Q Zhang Y . Tgf-beta1 secreted by M2 phenotype macrophages enhances the stemness and migration of glioma cells via the Smad2/3 signalling pathway. Int J Mol Med. (2018) 42:3395–403. doi: 10.3892/ijmm.2018.3923 30320350 PMC6202079

[118] Zhang G Tao X Ji B Gong J . Hypoxia-driven M2-polarized macrophages facilitate cancer aggressiveness and temozolomide resistance in glioblastoma. Oxid Med Cell Longev. (2022) 2022:1614336. doi: 10.1155/2022/1614336 36046687 PMC9423979

[119] Yao Y Ye H Qi Z Mo L Yue Q Baral A . B7-H4(B7x)-mediated cross-talk between glioma-initiating cells and macrophages via the Il6/Jak/Stat3 pathway lead to poor prognosis in glioma patients. Clin Cancer Res. (2016) 22:2778–90. doi: 10.1158/1078-0432.CCR-15-0858 27001312 PMC4891287

[120] Lin L Cai J Jiang C . Recent advances in targeted therapy for glioma. Curr Med Chem. (2017) 24:1365–81. doi: 10.2174/0929867323666161223150242 28019637

[121] Zhao L Qiu Z Yang Z Xu L Pearce TM Wu Q . Lymphatic endothelial-like cells promote glioblastoma stem cell growth through cytokine-driven cholesterol metabolism. Nat Cancer. (2024) 5:147–66. doi: 10.1038/s43018-023-00658-0 38172338

[122] Seerapu VN Amaresan R Gopal U . From integrated analysis to clinical insight: Ncrna-mediated ferroptosis in glioblastoma. Cancers (Basel). (2026) 18. doi: 10.3390/cancers18081238 42073563 PMC13115343

[123] Tsvetkov P Coy S Petrova B Dreishpoon M Verma A Abdusamad M . Copper induces cell death by targeting lipoylated Tca cycle proteins. Science. (2022) 375:1254–61. doi: 10.1126/science.abf0529 35298263 PMC9273333

[124] Huang Y Su D Cui X Ding Y Zhan Q Hong B . Neu1 sustains mitochondria-lysosome contacts to trigger lysosomal Cu(2)+/cathepsin escape and mitochondrial lysis in glioblastoma (Neulysis). Neuro Oncol. (2026) 28:1662–79. doi: 10.1093/neuonc/noag087 42015369 PMC13338325

[125] Feng W Yang R Weng C Li C Shi W Chen Y . Reversing temozolomide resistance in glioblastoma based on cuproptosis-mediated positive feedback loop. Adv Sci (Weinh). (2026) 13:e76677. doi: 10.1002/advs.76677 42474148 PMC13383701

[126] Hardee ME Marciscano AE Medina-Ramirez CM Zagzag D Narayana A Lonning SM . Resistance of glioblastoma-initiating cells to radiation mediated by the tumor microenvironment can be abolished by inhibiting transforming growth factor-beta. Cancer Res. (2012) 72:4119–29. doi: 10.1158/0008-5472.CAN-12-0546 22693253 PMC3538149

[127] Liu Q Palomero L Moore J Guix I Espin R Aytes A . Loss of Tgfbeta signaling increases alternative end-joining DNA repair that sensitizes to genotoxic therapies across cancer types. Sci Transl Med. (2021) 13. doi: 10.1126/scitranslmed.abc4465 33568520 PMC8208885

[128] Li D Cui G Yang K Lu C Jiang Y Zhang L . Inhibiting macrophage-derived lactate transport restores Cgas-Sting signalling and enhances antitumour immunity in glioblastoma. Nat Cell Biol. (2026) 28:349–62. doi: 10.1038/s41556-025-01839-y 41495200

[129] Wang L Zhen T Liu H Sun P Wu X Chen Y . Rewiring cell death and evading repair: Evolution of temozolomide and emerging strategies against resistant glioblastoma. Eur J Med Chem. (2026) 316:119049. doi: 10.1016/j.ejmech.2026.119049 42314466

[130] Guo Y Guo J Xiao W Lu Y . Role of non-coding Rnas in O(6)-methylguanine-DNA methyltransferase-positive glioblastoma (Review). Oncol Lett. (2026) 31:195. doi: 10.3892/ol.2026.15550 41947894 PMC13051283

[131] Gupta SK Jain S Friedman TR Sarkaria JN . Divergent roles of canonical and non-canonical mismatch repair in regulating temozolomide sensitivity in glioblastoma. Int J Mol Sci. (2026) 27. doi: 10.3390/ijms27146517 42511857 PMC13410232

[132] Radtke L Majchrzak-Celinska A Awortwe C Vater I Nagel I Sebens S . Crispr/Cas9-induced knockout reveals the role of Abcb1 in the response to temozolomide, carmustine and lomustine in glioblastoma multiforme. Pharmacol Res. (2022) 185:106510. doi: 10.1016/j.phrs.2022.106510 36252775

[133] Lopez-Lopez S Castro-Robles B Garcia-Flores N Diaz-Guerra MJM Arias-Salazar L Sandoval H . Proinflammatory macrophage secretome enhances temozolomide sensitivity in glioblastoma via Pstat3-mediated downregulation of DNA repair enzymes. Sci Rep. (2025) 15:38875. doi: 10.1038/s41598-025-22674-z 41198755 PMC12592360

[134] Jahangiri A Nguyen A Chandra A Sidorov MK Yagnik G Rick J . Cross-activating C-Met/Beta1 integrin complex drives metastasis and invasive resistance in cancer. Proc Natl Acad Sci USA. (2017) 114:E8685–94. doi: 10.1073/pnas.1701821114 28973887 PMC5642678

[135] Piao Y Liang J Holmes L Zurita AJ Henry V Heymach JV . Glioblastoma resistance to anti-Vegf therapy is associated with myeloid cell infiltration, stem cell accumulation, and a mesenchymal phenotype. Neuro Oncol. (2012) 14:1379–92. doi: 10.1093/neuonc/nos158 22965162 PMC3480262

[136] Min HK Zhao H Bahcheli A Pan P Chan J Chen J . Fluid shear stress activates a targetable mechano-metastatic cascade to promote medulloblastoma metastasis. Nat BioMed Eng. (2026) 10:565–83. doi: 10.1038/s41551-025-01487-5 40897897

[137] Ahmady F Curpen P Perriman L Fonseca Teixeira A Wu S Zhu HJ . Reduced T and Nk cell activity in glioblastoma patients correlates with Tim-3 and Bat3 dysregulation. Cells. (2024) 13. doi: 10.3390/cells13211777 39513882 PMC11545661

[138] Mukherjee A . Immune checkpoint crosstalk between Lag-3 and Cd39/Cd73 in glioblastoma: Dual-pathway regulation of metabolic exhaustion and therapeutic reversal strategies. J Egypt Natl Canc Inst. (2026) 38. doi: 10.1186/s43046-026-00361-y 42289542 PMC13313299

[139] Xiao L Hu R Chen W Gao J Zhao Y Wang Z . An antibody targeting an immune checkpoint molecule Btn2a2 enhances anti-tumor immunity. Neoplasia. (2025) 65:101161. doi: 10.1016/j.neo.2025.101161 40262436 PMC12053764

[140] Zhai L Lauing KL Chang AL Dey M Qian J Cheng Y . The role of Ido in brain tumor immunotherapy. J Neuro-Oncol. (2015) 123:395–403. doi: 10.1007/s11060-014-1687-8 25519303 PMC4641522

[141] Palange AL Guerriero I Spano R Susana A Lopez-Estevez AM Furnari AG . Acd47-docetaxel immuno-chemotherapy: A two-pronged attack against 'cold' glioblastoma. J Control Release. (2026) 398:115194. doi: 10.1016/j.jconrel.2026.115194 42486280

[142] Lee GA Hsu JB Chang YW Hsieh LC Li YT Wu YC . Il-19 as a promising theranostic target to reprogram the glioblastoma immunosuppressive microenvironment. J BioMed Sci. (2025) 32:34. doi: 10.1186/s12929-025-01126-w 40057744 PMC11889942

[143] Saltis L Mun LJ . Targeting T-cell exhaustion and tumour immune evasion: A review of emerging strategies. Pathol Res Pract. (2026) 287:156638. doi: 10.1016/j.prp.2026.156638 42561529

[144] Wick A Desjardins A Suarez C Forsyth P Gueorguieva I Burkholder T . Phase 1b/2a study of galunisertib, a small molecule inhibitor of transforming growth factor-beta receptor I, in combination with standard temozolomide-based radiochemotherapy in patients with newly diagnosed malignant glioma. Invest New Drugs. (2020) 38:1570–9. doi: 10.1007/s10637-020-00910-9 32140889 PMC7497674

[145] Yan D Kowal J Akkari L Schuhmacher AJ Huse JT West BL . Inhibition of colony stimulating factor-1 receptor abrogates microenvironment-mediated therapeutic resistance in gliomas. Oncogene. (2017) 36:6049–58. doi: 10.1038/onc.2017.261 28759044 PMC5666319

[146] Butowski N Colman H De Groot JF Omuro AM Nayak L Wen PY . Orally administered colony stimulating factor 1 receptor inhibitor Plx3397 in recurrent glioblastoma: An Ivy Foundation Early Phase Clinical Trials Consortium phase II study. Neuro Oncol. (2016) 18:557–64. doi: 10.1093/neuonc/nov245 26449250 PMC4799682

[147] Cai W Hu J Chen C Shi H Xu L Zhu S . Thr3/Ser90 phosphorylation-stabilized S100a8 regulates cholesterol metabolism in glioblastoma stem cells. Oncogene. (2026) 45:2120–36. doi: 10.1038/s41388-026-03799-5 42009969

[148] Ahmad F Rendina BP Chen C Ren H Brantner C Dukic T . Systemic cyst(e)inase administration induces ferroptosis and synergizes with temozolomide in glioblastoma. iScience. (2026) 29:114350. doi: 10.1016/j.isci.2025.114350 41541699 PMC12799779

[149] Zhu Z Zhai Y Hao Y Wang Q Han F Zheng W . Specific anti-glioma targeted-delivery strategy of engineered small extracellular vesicles dual-functionalised by Angiopep-2 and Tat peptides. J Extracell Vesicles. (2022) 11:e12255. doi: 10.1002/jev2.12255 35932288 PMC9451528

[150] Reardon DA Brandes AA Omuro A Mulholland P Lim M Wick A . Effect of nivolumab vs bevacizumab in patients with recurrent glioblastoma: The Checkmate 143 phase 3 randomized clinical trial. JAMA Oncol. (2020) 6:1003–10. doi: 10.1001/jamaoncol.2020.1024 32437507 PMC7243167

[151] Omuro A Brandes AA Carpentier AF Idbaih A Reardon DA Cloughesy T . Radiotherapy combined with nivolumab or temozolomide for newly diagnosed glioblastoma with unmethylated Mgmt promoter: An international randomized phase iii trial. Neuro Oncol. (2023) 25:123–34. doi: 10.1093/neuonc/noac099 35419607 PMC9825306

[152] Lim M Weller M Idbaih A Steinbach J Finocchiaro G Raval RR . Phase iii trial of chemoradiotherapy with temozolomide plus nivolumab or placebo for newly diagnosed glioblastoma with methylated Mgmt promoter. Neuro Oncol. (2022) 24:1935–49. doi: 10.1093/neuonc/noac116 35511454 PMC9629431

[153] Li Y He X Zhou H Tian Y Zhang Q Zhou Y . Clinical advances in Cd47-Sirpalpha axis-targeted cancer immunotherapy: Mechanisms, strategies, challenges, and future perspectives. Biochem Biophys Res Commun. (2026) 802:153330. doi: 10.1016/j.bbrc.2026.153330 41579727

[154] Ku KB Kim CW Kim Y Kang BH La J Kang I . Inhibitory Fcgamma receptor deletion enhances Cd8 T cell stemness increasing anti-Pd-1 therapy responsiveness against glioblastoma. J Immunother Cancer. (2024) 12. doi: 10.1136/jitc-2024-009449 39461881 PMC11529582

[155] O'Rourke DM Nasrallah MP Desai A Melenhorst JJ Mansfield K Morrissette JJD . A single dose of peripherally infused Egfrviii-directed Car T cells mediates antigen loss and induces adaptive resistance in patients with recurrent glioblastoma. Sci Transl Med. (2017) 9. doi: 10.1126/scitranslmed.aaa0984 28724573 PMC5762203

[156] Brown CE Hibbard JC Alizadeh D Blanchard MS Natri HM Wang D . Locoregional delivery of Il-13ralpha2-targeting Car-T cells in recurrent high-grade glioma: A phase 1 trial. Nat Med. (2024) 30:1001–12. doi: 10.1038/s41591-024-02875-1 38454126 PMC11031404

[157] Choi BD Gerstner ER Frigault MJ Leick MB Mount CW Balaj L . Intraventricular Carv3-Team-E T cells in recurrent glioblastoma. N Engl J Med. (2024) 390:1290–8. doi: 10.1056/NEJMoa2314390 38477966 PMC11162836

[158] Bagley SJ Logun M Fraietta JA Wang X Desai AS Bagley LJ . Intrathecal bivalent Car T cells targeting Egfr and Il13ralpha2 in recurrent glioblastoma: Phase 1 trial interim results. Nat Med. (2024) 30:1320–9. doi: 10.1038/s41591-024-02893-z 38480922 PMC13123313

[159] Zhai Y Li G Pan C Yu M Hu H Wang D . The development and potent antitumor efficacy of Cd44/Cd133 dual-targeting Il7ralpha-armored Car-T cells against glioblastoma. Cancer Lett. (2025) 614:217541. doi: 10.1016/j.canlet.2025.217541 39952598

[160] Poorva P Mast J Cao B Shah MV Pollok KE Shen J . Killing the killers: Natural killer cell therapy targeting glioma stem cells in high-grade glioma. Mol Ther. (2025) 33:2462–78. doi: 10.1016/j.ymthe.2025.02.043 40040281 PMC12172185

[161] Zannikou M Duffy JT Levine RN Seblani M Liu Q Presser A . Il15 modification enables Car T cells to act as a dual targeting agent against tumor cells and myeloid-derived suppressor cells in Gbm. J Immunother Cancer. (2023) 11. doi: 10.1136/jitc-2022-006239 36759014 PMC9923337

[162] Yueh PF Chiang IT Weng YS Liu YC Wong RCB Chen CY . Innovative dual-gene delivery platform using Mir-124 and Pd-1 via umbilical cord mesenchymal stem cells and exosome for glioblastoma therapy. J Exp Clin Cancer Res. (2025) 44:107. doi: 10.1186/s13046-025-03336-4 40134003 PMC11934454

[163] Figg JW Love C Stansbury S Jin D Francis C Dean BD . Adoptive cellular therapy prevents reconstitution of myeloid-derived suppressor cells in the glioma tumor microenvironment. Neuro-Oncol Adv. (2026) 8:vdag054. doi: 10.1093/noajnl/vdag054 41908149 PMC13019303

[164] Lo T Bunjaj A Turnbull JP Desai S Alsalahi A Heitkotter H . Glioblastoma as a neuro-immune network disorder: Rethinking the tumor microenvironment, neural circuit integration, and therapeutic resistance. Oncol Rev. (2026) 20:1906725. doi: 10.3389/or.2026.1906725 42577360 PMC13454049

[165] Marampon F Leoni F Mancini A Pietrantoni I Codenotti S Ferella L . Histone deacetylase inhibitor Itf2357 (Givinostat) reverts transformed phenotype and counteracts stemness in *in vitro* and *in vivo* models of human glioblastoma. J Cancer Res Clin Oncol. (2019) 145:393–409. doi: 10.1007/s00432-018-2800-8 30474756 PMC11810183

[166] Doualle C Guerin M Codron P Spinazzi M Tessier A Lelievre B . Ezh2 inhibition in resistant glioblastoma stem cells counteracts oxidative stress protection and triggers ferroptosis. BioMed Pharmacother. (2026) 198:119254. doi: 10.1016/j.biopha.2026.119254 41861524

[167] Sidhu RS Guo S Wang G Liu M . Role of Notch and its oncogenic signaling crosstalk in glioma and glioma stem cells. Gene. (2025) 969:149761. doi: 10.1016/j.gene.2025.149761 40935208 PMC13502891

[168] Han F Xu Y Qian C Meng B Mo X Chen Y . (18)F-Fdg Pet imaging reveals Gw4869's suppression of glioblastoma malignant progression and reversal of Tmz chemoresistance via glucose metabolic phenotype remodeling. J Transl Med. (2026) 24:245. doi: 10.1186/s12967-025-07668-9 41566316 PMC12905924

[169] Kim J Kim M Han H Kim S Lahiji SF Kim YH . Dual-delivery of exosome inhibitor and immune-activating gene via lipid nano-assemblies for tumor immune evasion inhibition. J Control Release. (2025) 381:113569. doi: 10.1016/j.jconrel.2025.02.065 39993637

[170] Agosti E Antonietti S Ius T Fontanella MM Zeppieri M Panciani PP . A systematic review of mesenchymal stem cell-derived extracellular vesicles: A potential treatment for glioblastoma. Brain Sci. (2024) 14. doi: 10.3390/brainsci14111058 39595821 PMC11591642

[171] Ramachandran M Vaccaro A van de Walle T Georganaki M Lugano R Vemuri K . Tailoring vascular phenotype through Aav therapy promotes anti-tumor immunity in glioma. Cancer Cell. (2023) 41:1134–51.e10. doi: 10.1016/j.ccell.2023.04.010 37172581

